# Endo‐Lysosomal Network Disorder Reprograms Energy Metabolism in *SorL1*‐Null Rat Hippocampus

**DOI:** 10.1002/advs.202407709

**Published:** 2024-09-03

**Authors:** Yajie Wang, Yuting Yang, Ying Cai, Ayikaimaier Aobulikasimu, Yuexin Wang, Chuanwei Hu, Zhikang Miao, Yue Shao, Mengna Zhao, Yue Hu, Chang Xu, Xinjun Chen, Zhiqiang Li, Jincao Chen, Lianrong Wang, Shi Chen

**Affiliations:** ^1^ Department of Gastroenterology Hubei Clinical Center and Key Laboratory of Intestinal and Colorectal Disease Zhongnan Hospital of Wuhan University School of Pharmaceutical Sciences Wuhan University Wuhan Hubei 430071 China; ^2^ Department of Respiratory Diseases, Institute of Pediatrics Shenzhen Children's Hospital Shenzhen 518026 China; ^3^ Department of Burn and Plastic Surgery Shenzhen Key Laboratory of Microbiology in Genomic Modification & Editing and Application Shenzhen Institute of Translational Medicine Medical Innovation Technology Transformation Center Shenzhen University Medical School, Shenzhen Second People's Hospital The First Affiliated Hospital of Shenzhen University Shenzhen 518035 China; ^4^ Brain Center, Department of Neurosurgery, Ministry of Education Key Laboratory of Combinatorial Biosynthesis and Drug Discovery TaiKang Center for Life and Medical Sciences, Zhongnan Hospital of Wuhan University, School of Pharmaceutical Sciences, Wuhan University Wuhan 430071 China

**Keywords:** Alzheimer's disease, endo‐lysosome network disorder, energy metabolism, hippocampus degeneration, iron homeostasis, *SorL1*

## Abstract

*Sortilin‐related receptor 1* (*SorL1*) deficiency is a genetic predisposition to familial Alzheimer’s disease (AD), but its pathology is poorly understood. In *SorL1*‐null rats, a disorder of the global endosome‐lysosome network (ELN) is found in hippocampal neurons. Deletion of *amyloid precursor protein* (*APP*) in *SorL1*‐null rats could not completely rescue the neuronal abnormalities in the ELN of the hippocampus and the impairment of spatial memory in *SorL1*‐null young rats. These in vivo observations indicated that APP is one of the cargoes of *SorL1* in the regulation of the ELN, which affects hippocampal‐dependent memory. When *SorL1* is depleted, the endolysosome takes up more of the lysosome flux and damages lysosomal digestion, leading to pathological lysosomal storage and disturbance of cholesterol and iron homeostasis in the hippocampus. These disturbances disrupt the original homeostasis of the material‐energy‐subcellular structure and reprogram energy metabolism based on fatty acids in the *SorL1*‐null hippocampus, instead of glucose. Although fatty acid oxidation increases ATP supply, it cannot reduce the levels of the harmful byproduct ROS during oxidative phosphorylation, as it does in glucose catabolism. Therefore, the *SorL1*‐null rats exhibit hippocampal degeneration, and their spatial memory is impaired. Our research sheds light on the pathology of *SorL1* deficiency in AD.

## Graphical abstract

Glucose is the primary carbon source in the mammalian brain. But in *SorL1*‐null rat hippocampus, the global endosome‐lysosome network is disrupted, allowing fatty acids instead of glucose as the main carbon source, namely the reprogramming of material‐energy metabolism. Although fatty acid oxidation efficiently generates ATP, it cannot neutralize ROS. Therefore, the SorL1‐null rats exhibit hippocampal degeneration. ‐

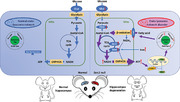

## 1 Introduction

In 2007, haplotype insufficiency of Sortilin‐related receptor 1 (*SorL1*) was found to increase the risk of Alzheimer's disease (AD).^[^ ^1^ ^]^ Exome sequencing revealed that *SorL*1 coding variants are closely associated with early‐onset familial AD.^[^ ^2^ ^]^ Mutations with coding sequence truncation occurred exclusively in AD patients,^[^ ^2^, ^3^ ^]^ confirming the role of *SorL1* as a critical protector against AD.

SorL1 was identified as a sorting receptor belonging to the low‐density lipoprotein (LDL) family^[^ ^4^ ^]^ and is highly expressed in the hippocampal neurons of the central nervous system in mice and humans.^[^ ^5^ ^]^ SorL1 interacts directly with amyloid precursor protein (APP)^[^ ^6^ ^]^ and is responsible for the reverse transport of APP from the endosome to the trans‐Golgi network (TGN),^[^ ^7^ ^]^ thereby facilitating APP relocalization to the plasma membrane and reducing amyloid‐beta plaque (Aβ) generation. SorL1 traffics not only APP but also brain‐derived neurotrophic factor (BDNF)^[^ ^8^ ^]^ and glial‐derived neurotrophic factor (GDNF).^[^ ^9^ ^]^ This molecule also impacts synapsin^[^ ^10^ ^]^ and erythropoietin‐producing hepatocellular carcinoma A4 (EphA4),^[^ ^11^ ^]^ the synaptic regulators of neurotransmitter release and synaptic structure, respectively. These known functions of SorL1 support the assertion in genetic research that this protein is an important factor in the pathogenesis of AD. Furthermore, the role of SorL1 in AD is not limited to the regulation of the processing of APP to Aβ, and this protein likely has additional pathological functions related to AD. Recent studies have linked SorL1 to endosomal dysfunction, another pathological feature of AD.^[^ ^12^ ^]^ Subsequently, two studies on the effect of *SorL1* deletion on human induced pluripotent stem cells (iPSCs) were published, both of which demonstrated that *SorL1*‐deficient neurons undergo significant endosomal enlargement (EE).^[^ ^13^ ^]^ Pathological EE has been demonstrated in the *SorL1* haploinsufficiency minipig model,^[^ ^14^ ^]^ but the pathology of *SorL1* deficiency remains unclear.

EE in AD brain neurons is an early cytopathological hallmark that occurs decades prior to the appearance of Aβ aggregates.^[^ ^12^ ^]^ This finding indicates delayed maturation of endosomes or blockage of the endocytic network and is a sign of endosomal‐lysosome dysfunction.^[^ ^15^ ^]^ The endo‐lysosomal network (ELN) consists of endosomes and lysosomes, which are membranous organelles that interconvert in a fluid and dynamic manner. The ELN is essential for the transport, sorting, degradation, and redistribution of various substances, including proteins,^[^ ^16^ ^]^ ions,^[^ ^17^ ^]^ and lipids.^[^ ^18^ ^]^ In the ELN, endosomes shuttle between the lysosome and other membranous organelles, while lysosomes are mainly responsible for the degradation of biomolecules and subcellular structures and therefore serve as metabolic sensing signal hubs.^[^ ^19^ ^]^ Cholesterol is a major component of cell membranes, and its intracellular dispersion is mediated by lysosomes through interactions with the endoplasmic reticulum (ER), peroxisomes, TGN, and mitochondria.^[^ ^20^ ^]^ When endocytosis is mediated by the LDL‐receptor, cholesterol esters are delivered to the limiting membrane of the lysosome. In lysosomes, LDL‐derived cholesterol esters are hydrolyzed to free cholesterol by lysosomal acid lipase.^[^ ^21^ ^]^ Most free cholesterol is immediately bound by proteins with hydrophobic sterol‐binding domains for transport out of the lysosome. Lysosomal associated membrane protein (Lamp) proteins, including Lamp1 and Lamp2, directly bind cholesterol with high affinity and participate in the exit of cholesterol from lysosomes.^[^ ^22^, ^23^ ^]^

Lysosomes are also the controlling centers of cellular iron transport and homeostasis. Extracellular Fe^3+^ enters the lysosome via the ELN and is reduced to Fe^2+^ in the acidic lumen of late endosomes and lysosomes.^[^ ^17^ ^]^ The activity of the enzyme that catalyzes this process depends on the acidic pH of the organelles.^[^ ^24^ ^]^ Reduced iron is released by the lysosome into the cytoplasmic labile iron pool (LIP) and is ready to be transferred or utilized to different cell compartments. Mitochondria are the main sites of iron utilization for the biosynthesis of heme and iron‐sulfur (Fe‐S) clusters, the latter of which are cofactor of most respiration chain complexes.^[^ ^17^ ^]^ Thus, lysosome‐mediated regulation of iron homeostasis is also involved in mitochondrial energy production. Due to the high reactivity of Fe^2+^, excess free Fe^2+^ will generate hydrogen peroxide (H_2_O_2_) in the Fenton reaction to destroy membrane lipids and other biomacromolecules, triggering cell death.^[^ ^25^ ^]^

The understanding of pathological lysosomal storage has recently improved. Substrate accumulation in lysosomes can lead to pathogenic downstream cascades, including mitochondrial dysfunction.^[^ ^26^ ^]^ This phenomenon is because healthy mitochondrial homeostasis is maintained by the removal and degradation of damaged mitochondria from the network through lysosomal‐mediated mitophagy.^[^ ^27^ ^]^ When lysosomes are impaired, mitochondrial function is also affected. Conversely, damaged mitochondria reduce the adenosine 5' triphophate (ATP) supply that sustains the acidic digestion environment of lysosomes, which also leads to lysosomal dysfunction.^[^ ^28^ ^]^ In this way, efficient crosstalk and interaction between lysosomes and mitochondria must always be maintained. This consensus has been confirmed in a variety of lysosomal storage diseases (LSDs), such as Gaucher disease, type C Niemann‐Pick disease, and Krabbe disease,^[^ ^26^ ^]^ which have also been proposed as potential mechanisms of neurodegeneration.^[^ ^29^, ^30^ ^]^

EE was observed in *SorL1*‐null iPSCs and haploinsufficient minipigs, suggesting that the underexpression of SorL1 leads to ELN dysfunction and lysosome impairment, which remains to be evaluated. Therefore, we generated a *SorL1*‐null rat line and studied the cytopathology of *SorL1* deficiency in the hippocampus.

## 2 Results

### 2.1 APP Depletion Could Not Completely Rescue *SorL1*‐Null Induced Endosome‐Lysosome Abnormalities in the Rat Hippocampus

**Figure 1:**
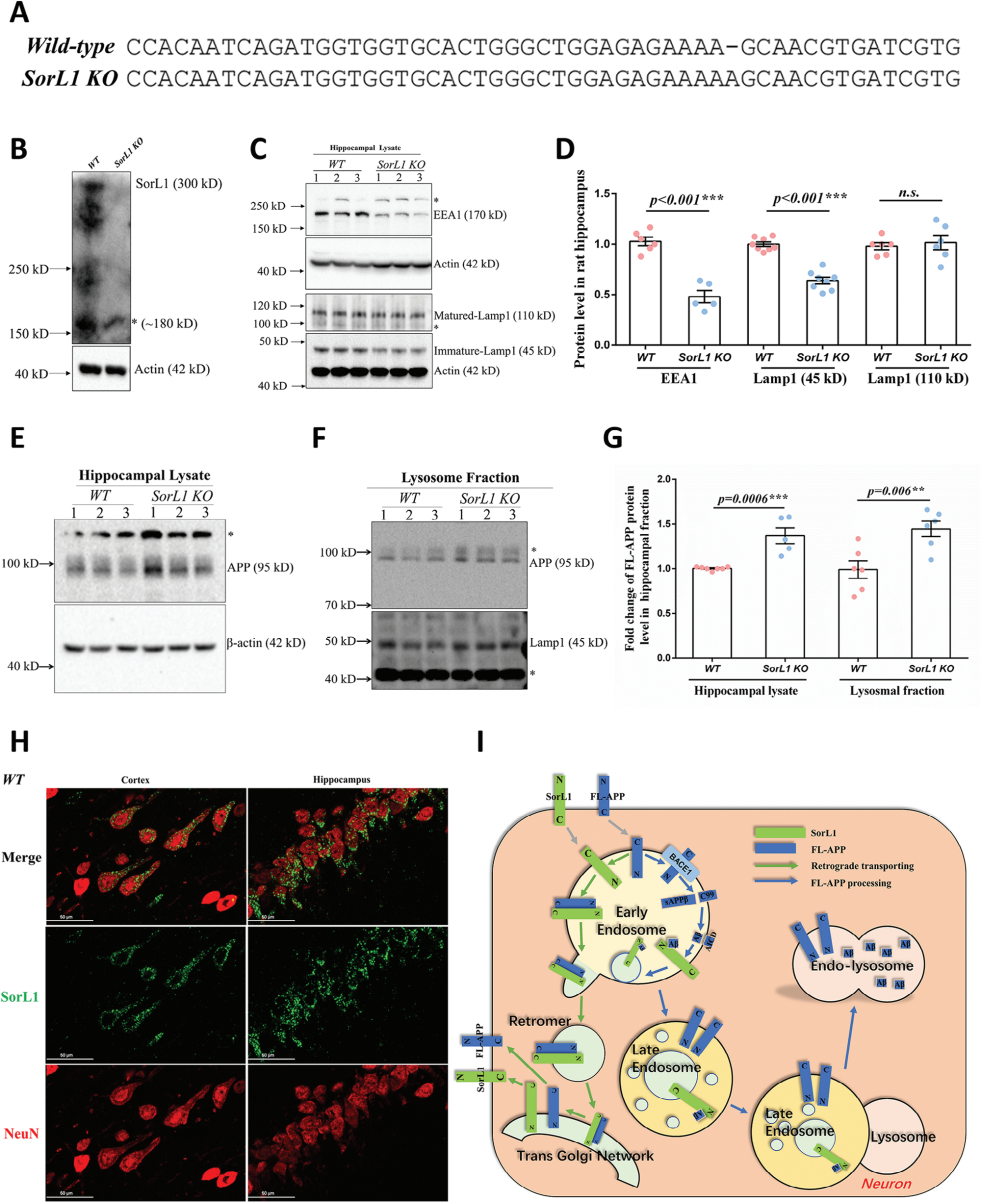
Accumulation of full‐length APP in the ELN of the *SorL1*‐null hippocampus. A) W*ild‐type* and mutated *SorL1* genes were sequenced at the TALEN targeting site in the rat genome. One base pair insertion led to an open reading frame (ORF) shift in exon 2 of the *SorL1* gene. B) Western blot assay verified the complete absence of the SorL1 protein in the hippocampus of mutant rats (*SorL1 KO*), and *wild‐type* rats (*WT*) were used as the positive control. A mixed sample of three male rats from each genotype was detected. C) Protein levels of EEA1 and Lamp1 were measured in hippocampal lysates from three *WT* and *SorL1 KO* rats. β‐Actin was used as the loading control. The hippocampus was dissected by dissection from 3‐month‐old male rats. The molecular weight of each protein is provided on the right, and the stray band is indicated by the asterisk. D) Data from individual rats are shown by dots, and the mean ± SEM values are presented in a bar chart. Three independent experiments were performed. For the EEA1 assay, *WT* (*n* = 6) and *SorL1 KO* (*n* = 5) rats were calculated. For the Lamp1 assay, *WT* (*n* = 8) and *SorL1 KO* (*n* = 8) rats were detected. E,F) Protein levels of full‐length APP in the hippocampal lysates and lysosomal fraction were detected. An equal amount of protein was loaded in each lane. The stray band is indicated by the asterisk. G) Data from individual rats are indicated by dots, and the mean ± SEM values are presented in a bar chart. Three independent experiments were performed. For the hippocampal lysate assay, *WT* (*n* = 7) and *SorL1 KO* (*n* = 5) rats were analyzed. For the lysosomal fraction assay, *WT* (*n* = 6) and *SorL1 KO* (*n* = 6) rats were analyzed. Two‐tailed *t*‐tests were performed, with ^**^ *p* < 0.01 and ^***^ *p* < 0.001 indicating significant differences. H) Neuronal immunoreactivity of SorL1 in the rat brain. Neurons were immunostained with an anti‐NeuN antibody (red), and SorL1 in the cortex and hippocampus of *WT* rats was immunostained with an anti‐SorL1 antibody (green). Scale bar = 50 µm. Three‐month‐old male rats were used for sample collection in (B‐H). I) SorL1‐mediated intracellular APP sorting in neurons. In early endosomes (EEs), SorL1 binds to APP to facilitate its retrograde transport from EEs to the trans‐Golgi network (TGN), which inhibits β‐secretase cleavage of APP by beta‐site amyloid precusor protein cleaving enzyme 1 (BACE1) on endosomes. Unprocessed full‐length APP can be reserved on the endosomal membrane until EEs mature into late endosomes (LEs) and fuse with lysosomes to form endolysosomes. SorL1 binds to Aβ in the EE lumen, mediating its sorting into LEs for endolysosomal degradation. N, N‐terminus of the protein; C, C‐terminus of the protein.

To investigate the pathology of *SorL1* deficiency in the central nervous system, the *SorL1* gene was targeted to generate *SorL1*‐null (*SorL1 KO*) rats through a transcription activator‐like effector nuclease (TALEN). One base pair insertion in exon 2 was found in the offspring of the chimeras through sequencing (**Figure** **1**A). The hippocampus was then collected to determine the abundance of the SorL1 protein in the lysates of *SorL1 KO* homozygous rat tissues, in which the SorL1 protein was completely absent (Figure 1B). Early EE and lysosomal defects have been reported in *SorL1*‐deficient human iPSCs.^[^ ^13^ ^]^ To confirm the abnormalities in endosomes and lysosomes in vivo, the protein levels of early endosomal antigen 1 (EEA1) and lysosomal associated membrane proteint 1 (Lamp1), used as the indicators of ELN disorder, were measured in the hippocampal lysates of 3‐month‐old male *SorL1 KO* rats. Compared to those in *wild‐type* (*WT*) rats, the EEA1 and immature‐Lamp1 protein (45 kD) levels in *SorL1 KO* rats were reduced by approximately 52% and 36% (Figure 1C,D), respectively, suggesting that global ELN was disrupted by *SorL1* deficiency in the hippocampus, although the mature‐Lamp1 (110 kDa) appeared comparable between *WT* and *SorL1 KO* rats.

SorL1 mediates the intracellular sorting of APP, including retrograde transport from endosomes to the TGN, as well as the transport of Aβ from endosomes to lysosomes.^[^ ^7^ ^]^ These sorting routes are critical for inhibiting the processing of APP to Aβ, the neurotoxic hallmark of AD.^[^ ^31^ ^]^ We measured the abundance of full‐length APP in the *SorL1 KO* hippocampus and found that it was elevated by 36.9% (Figure 1E,G), indicating that the intrinsic disturbance of APP processing was caused by SorL1 depletion. On the endosomal membrane, SorL1 has been proven to bind full‐length APP and promote its retrograde transport, which could prevent APP from interacting with and being cleaved by the β‐secretase beta‐site amyloid precusor protein cleaving enzyme 1 (BACE1), the primary cleavage step for Aβ generation.^[^ ^32^ ^]^ Therefore, *SorL1* depletion results in the retention of full‐length APP on the endosome (Supplementary S1A–C, Supporting Information),^[^ ^1^, ^6^, ^33^ ^]^ where a portion of APP is sequentially cleaved by β/γ‐secretase, and the unprocessed APP remains on the endosome membrane until it matures and merges with the lysosome to form the endolysosomes. We then separated the lysosomal fraction from the rat hippocampus. The purity of the subcellular fractions was determined with Lamp1 for lysosomes, Histone 3 (H3) for the nucleus, and glyceraldehyde‐3‐phosphate dehydrogenase (GAPDH) for the cytoplasm (Supplementary S1D, Supporting Information). The full‐length APP content was indeed increased approximately 1.45‐fold in the hippocampal lysosomal fraction (Figure 1F,G). These results suggest that the loss of *SorL1* leads to the accumulation of full‐length APP in the rat hippocampal ELN rather than just Aβ generation.

**Figure 2:**
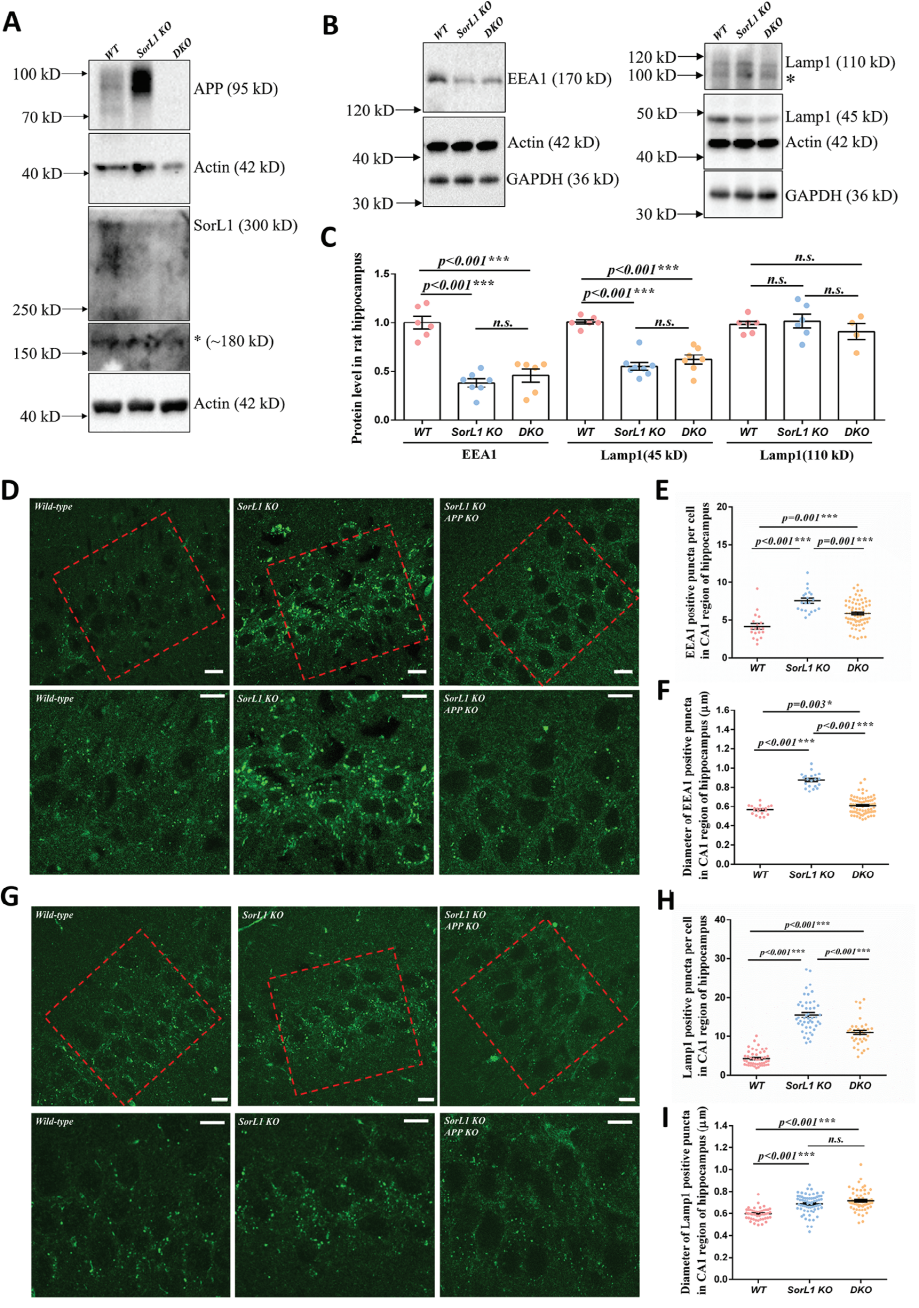
APP depletion cannot completely rescue ELN disorder in the *SorL1*‐null hippocampus. A) The *APP* gene was depleted in the *SorL1*‐deficient context through rat crossing. Hippocampal homogenates from *WT*, *SorL1 KO*, and *DKO* rats were analyzed for APP abundance. APP was not detected in the hippocampus of *DKO* rat. B,C) Protein levels of EEA1 and Lamp1 were measured in the hippocampal lysates of *WT*, *SorL1 KO*, and *DKO* rats. Three independent experiments of B) were executed. Data from individual rats are indicated by dots, and the mean ± SEM is presented in a bar chart in C). For the EEA1 assay, *WT* (*n* = 6), *SorL1 KO* (*n* = 7), and *DKO* (*n* = 6) rats were analyzed. For the Lamp1 assay, *WT* (*n* = 6), *SorL1 KO* (*n* = 8), and *DKO* (*n* = 7) rats were used. The stray band is indicated by the asterisk. D–I) Immunofluorescence staining and quantification of anti‐EEA1 and anti‐Lamp1 antibodies in brain slices from *WT*, *Sorl1 KO*, and *DKO* rats. Coronal slices from each rat were prepared, and the bilateral hippocampi were observed under a 60× objective. The CA1 region of the unilateral hippocampus was photographed and is presented in D) and G). The local area of the CA1 region in the hippocampus is indicated with a red square, and its enlarged image is presented underneath. Scale bar = 10 µm. For the count assay, the puncta in each photo were counted, and the counts were normalized to the neuron number. For diameter measurements, at least fifty clear‐edged puncta in each photo were measured and averaged. The index of each photo is indicated by a dot in E), F), H), and I). Three independent experiments involving 3‐month‐old male *WT* (*n* = 6), *SorL1 KO* (*n* = 6), and *DKO* (*n* = 6) rats were performed. Three‐month‐old male rats were used for sample collection. One‐way ANOVA was performed, and the differences are indicated by asterisks. ^*^ *p* < 0.05 and ^***^ *p* < 0.001 indicate significant differences, and *n.s*. indicates no significance.

The SorL1 protein is hyperactivated in neurons in mouse^[^ ^34^ ^]^ and pig^[^ ^14^ ^]^ brains. Therefore, we observed the localization of SorL1 in the rat cortex and hippocampus through coimmunostaining with neuronal nuclear protein (NeuN) and SorL1 antibodies. The merged signals that appeared in the *WT* rat brain suggested that SorL1 was primarily expressed in rat neurons (Figure 1H). Therefore, in *SorL1*‐deficient neurons, APP processing was promoted with the excessive deposition of its cleaved products, including the β‐cleavage products soluble APPbeta (sAPP_β_) and 99‐aa C‐terminal fragment of APP (C99), and then γ‐cleavage product Aβ, in endosomes and lysosomes (Figure 1I). All these cleavages could be considered responsible for EE and lysosomal dysfunction of *SorL1* deficiency. Furthermore, the elevated abundance of full‐length APP could also be responsible for the endosome‐lysosomal abnormalities in *SorL1*‐deficient neurons. To determine whether the accumulation of full‐length APP and its cleavages caused ELN disorder in the *SorL1‐*deficient hippocampus, we removed the *APP* gene from *SorL1 KO* rats through hybridization to generate *APP*‐*SorL1*‐null (*DKO*) rats, in which the APP protein was completely lost (**Figure** **2**A). The abundances of EEA1 and Lamp1 were compared among the *WT*, *SorL1 KO*, and *DKO* rats. In the *DKO* hippocampus, the abundance of EEA1 was comparable to that in the *SorL1 KO* rat hippocampus, and the abundance of EEA1 in both groups was lower than that in the *WT* rat hippocampus. Immature‐Lamp1 in the hippocampi of *DKO* and *SorL1 KO* rats were similar and lower than those in the hippocampi of *WT* rats (Figure 2B,C). In the *DKO* hippocampus, APP depletion did not restore EEA1 or Lamp1 levels to physiological levels comparable to those in *WT* rats, suggesting that the depletion of APP may not reverse *SorL1* deficiency‐induced endosome‐lysosome dysfunction.

Alterations in the protein abundance of EEA1 and Lamp1 in hippocampal lysates indicate that, at the expression level in hippocampal tissue, the abundance of endosomal‐ and lysosomal‐related proteins is adjusted to accommodate pathological changes caused by SorL1 defects, indicating that the endosomal‐ and lysosomal networks in the hippocampus are altered, but cannot reflect the intracellular counts and morphology of these organelles. The morphology of endosomes and lysosomes could be observed in neurons through EEA1 and Lamp1 immunostaining in the hippocampal CA1 region (Figure 2D,G). The number and diameter of EEA1‐positive puncta were measured and compared among *WT*, *SorL1 KO*, and *DKO* rats. The number of EEA1 puncta was approximately 4.2 in *WT* hippocampal neurons in brain slices, which was increased to 7.6 in *SorL1 KO* rats. In *DKO* rats, the number of EEA1 puncta was approximately 5.9, which was slightly greater than that in *WT* rats but lower than that in *SorL1 KO* rats (Figure 2E). The diameters of the EEA1 puncta exhibited a similar trend, with values of 0.57, 0.88, and 0.61 µm in the *WT*, *SorL1 KO*, and *DKO* neurons, respectively (Figure 2F). These data indicated that the presence of APP is not a unique pathology in *SorL1* deficiency‐induced EE. The number and diameter of Lamp1 puncta were also determined, and 4.3 Lamp1 puncta were detected per neuron in the hippocampus of the *WT* rats, with a diameter of 0.6 µm. In *SorL1 KO* neurons, the average Lamp1 puncta count was 15.5, and the average diameter was 0.69 µm, indicating lysosomal increase and enlargement. The number and diameter of Lamp1 puncta in the *DKO* hippocampus varied between *WT* and *SorL1 KO* rats, with *DKO* rats exhibiting 11 puncta and 0.72 µm in diameter (Figure 2H,I). *SorL1* deficiency results in early endosome enlargement with increased diameters and counts of EEA1‐positive puncta, and lysosome abnormalities with increased diameters and counts of Lamp1‐positive puncta in the CA1 region of the rat brain, indicating ELN disorder in neurons. If the disturbance of SorL1‐mediated APP processing is the only cause of ELN disorder in the context of *SorL1* deficiency, the abnormal morphology of early endosomes and lysosomes should be completely rescued by *APP* deletion in the *DKO* hippocampus, in which the endosomal and lysosomal morphology should be comparable to that in the *WT* hippocampus. In fact, the quantitative values of the diameter and counts of EEA1‐positive puncta in *DKO* brain slices were slightly better than *SorL1 KO* brain slices but still worse than those in the *WT* brain slices. Likewise, the extent of lysosome abnormalities in the *DKO* brain slices did not return to a physiological status equivalent to that of the *WT* brain slices. These results suggested that APP depletion cannot completely rescue the ELN abnormalities induced by *SorL1* deficiency in the hippocampus.

In the ELN, endosomes shuttle between lysosomes and other organelles of the intracellular membrane system, including the plasma membrane, Golgi, and ER, while lysosomes are the core organelles involved in intracellular digestion at the end of the ELN. The abnormal ELN morphology induced by *SorL1* deficiency could not be completely rescued by APP depletion, indicating that SorL1 is not only responsible for the intracellular sorting and processing of APP but also for global ELN regulation, especially in lysosomes.

### 2.2 Global Endosome‐Lysosome Impairment in the *SorL1*‐Null Hippocampus

**Figure 3:**
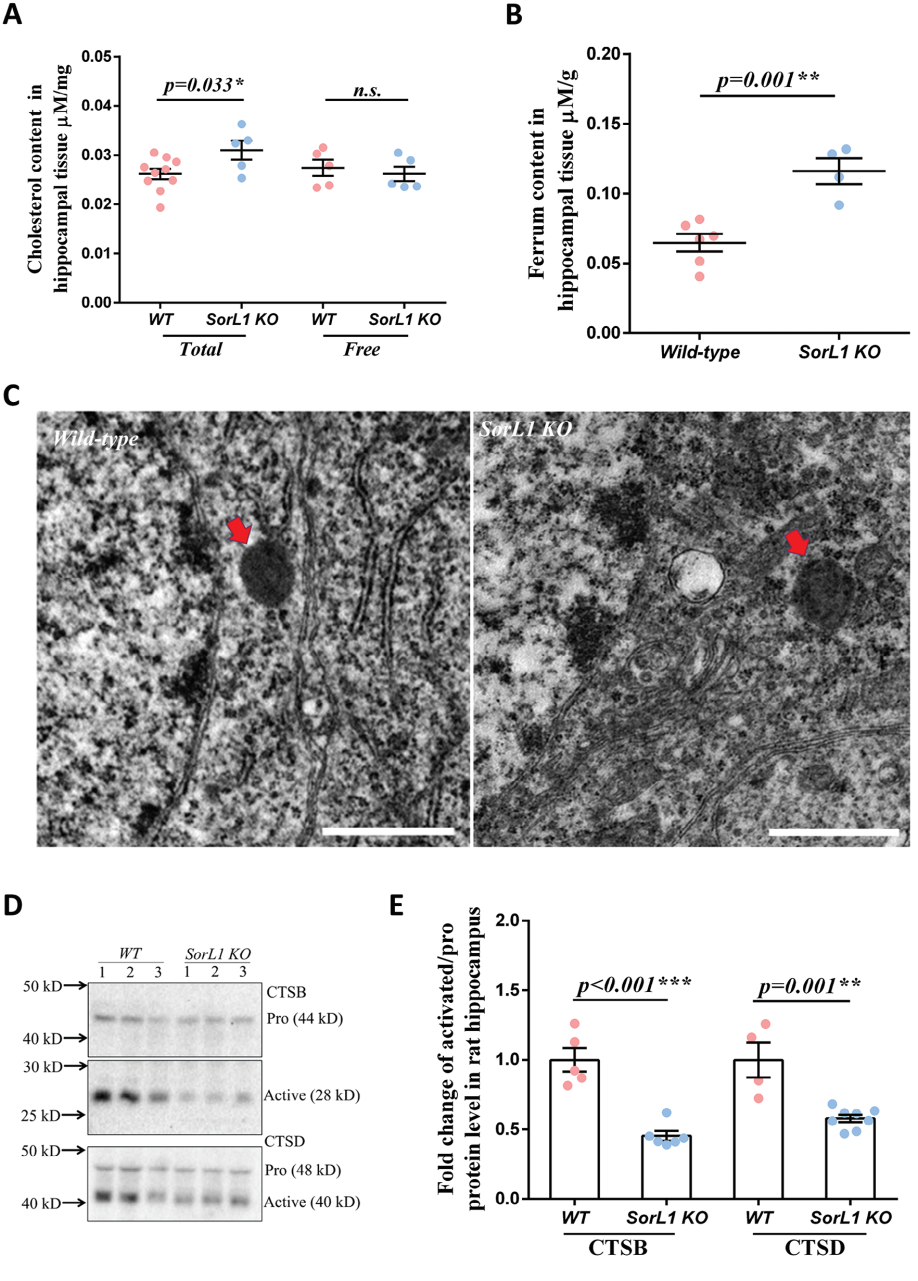
Global ELN disorder in the *SorL1*‐null hippocampus. A) Total and free cholesterol concentrations in the hippocampus of 3‐month‐old male rats. The values for individual rats are presented as dots. Three independent experiments with *WT* (*n* = 10) and *SorL1 KO* (*n* = 5) rats were performed for total cholesterol measurement, and *WT* (*n* = 5) and *SorL1 KO* (*n* = 5) rats were used for the free cholesterol assay. B) Tissue iron content in the hippocampus of 3‐month‐old male rats. The values from individual rats are presented as dots. Three independent experiments involving *WT* (*n* = 6) and *SorL1 KO* (*n* = 4) rats were performed. C) TEM images of pyramidal neurons in the hippocampus of 3‐month‐old male rats. Lysosomes are indicated by red arrows. Scale bar = 1 µm. D,E) Lysosomal digestive capacity was indicated by the activation of cathepsin B (CTSB) and D (CTSD). The protein levels of active/pro‐CTSB and active/pro‐CTSD were measured in the hippocampal lysate, as shown in D). The active‐/pro‐CTSB and active‐/pro‐CTSD ratios were calculated. The ratios from individual rats are indicated with dots in E), and the mean ± SEM values are presented in a bar chart. Three independent experiments were performed. For the CTSB assay, *WT* (*n* = 5) and *SorL1 KO* (*n* = 6) rats were used. For the CTSD assay, *WT* (*n* = 4) and *SorL1 KO* (*n* = 8) rats were used. Three‐month‐old male rats were used for sample collection. Two‐tailed *t*‐tests were performed, with ^*^p < 0.05, ^**^ *p* < 0.01, and ^***^ *p* < 0.001 indicating significant differences and *n.s*. indicating no significance.

Lysosomes receive and degrade endocytic and autophagosomal substances to meet the nutritional requirements of cells.^[^ ^35^ ^]^ Cholesterol, which is essential for cell membrane construction, enters endosomes through apolipoprotein receptor‐mediated endocytosis in neurons and is eventually degraded in lysosomes to release free cholesterol.^[^ ^18^ ^]^ To assess ELN uptake and cholesterol digestion in the *SorL1 KO* hippocampus, we evaluated total and free cholesterol levels in the tissue lysates. The total cholesterol content was approximately 0.026 µM per mg of tissue in the *WT* hippocampus, and the level increased by ≈20% (0.031 µm per mg of tissue) in the *SorL1 KO* hippocampus. However, the level of free cholesterol in the *SorL1 KO* hippocampus was comparable to that in the *WT* hippocampus (**Figure** **3**A). Given that SorL1 is primarily expressed in neurons (Figure 1H), these data suggested that ELN disruption alters the homeostasis of cholesteryl ester uptake and digestion in the *SorL1 KO* hippocampus. The ELN is also responsible for iron homeostasis regulation. Normally, iron is taken up through endocytosis, reduced in the ELN, and then released by the lysosome into the cytoplasm for further use by the cell.^[^ ^36^ ^]^ The hippocampal iron content was 1.81 times greater in *SorL1 KO* rats than in *WT* rats (Figure 3B), indicating that iron homeostasis is disrupted when SorL1 is absent in the hippocampus.

Both the degradation and distribution of cholesterol esters and iron reduction and release are mainly mediated by lysosomes. Thus, we distinguished lysosomes in hippocampal pyramidal neurons through transmission electron microscopy (TEM）. In *WT* neurons, the grayscale of lysosomes was uniform, and there were a few particles in the lumen, indicating their normal degradation ability. However, lysosomes in *SorL1 KO* neurons showed some distinct particles in the lumen, suggesting pathological lysosomal storage (Figure 3C). The lysosomal digestion capacity was measured through the activation of cathepsin B (CTSB) and cathepsin D (CTSD), both of which decreased in the *SorL1 KO* hippocampus, indicating impaired lysosomal digestion (Figure 3D,E).

**Figure 4:**
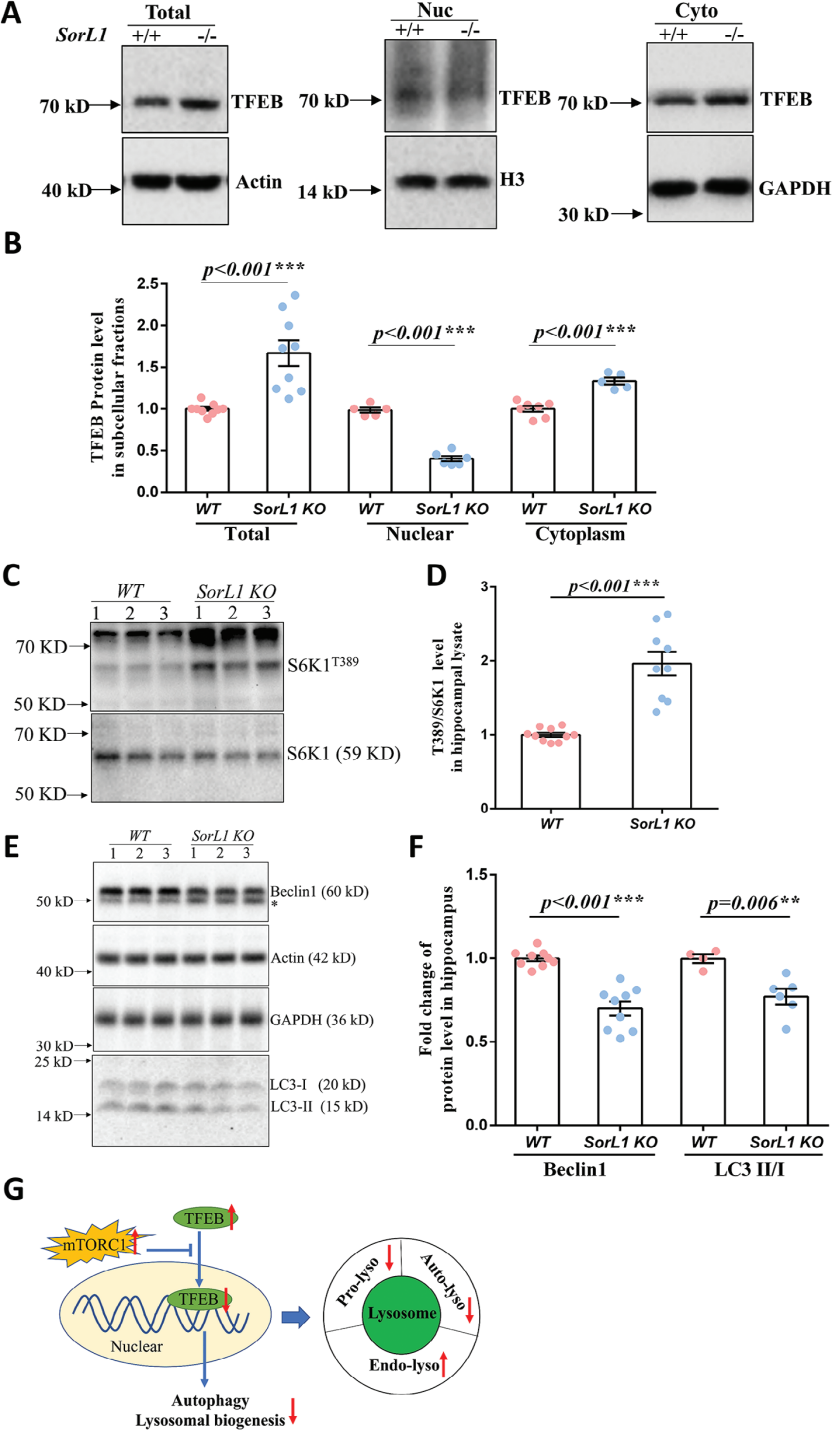
Endolysosomes account for most of the lysosome in the *SorL1*‐null hippocampus. A,B) Abundance of TFEB in the hippocampal lysate (total), nuclear (nuc), and cytoplasm (cyto) fractions are shown in (A). The TFEB protein levels of individual rats are presented by dots, and the bar chart represents the mean ± SEM in (B). Three independent experiments with hippocampal lysates from *WT* (*n* = 9) and *SorL1 KO* (*n* = 9) rats, *WT* (*n* = 5) and *SorL1 KO* (*n* = 6) rats in the nuclear fraction, and *WT* (*n* = 7) and *SorL1 KO* (*n* = 5) rats in the cytoplasmic fraction were performed. C,D) Activity of the mechanistic target of rapamycin complex 1 (mTORC1) in the rat hippocampus. Phosphorylation of S6K1 at T389 is regulated by mTORC1. The S6K1^T389^/S6K1 ratio was calculated in individual rats as shown in C), and the bar chart represents the mean ± SEM in D). Three independent experiments involving *WT* (*n* = 10) and *SorL1 KO* (*n* = 9) rats were performed. E,F) Protein levels of autophagy markers in the rat hippocampus. The stray band is indicated by the asterisk. Three independent experiments with *WT* (*n* = 9) and *SorL1 KO* (*n* = 9) rats were performed for the Beclin1 assay, and *WT* (*n* = 4) and *SorL1 KO* (*n* = 6) rats were performed for LC3 II/I ratio assessment. Three‐month‐old male rats were used for sample collection. Two‐tailed *t*‐tests were performed, with ^*^ *p* < 0.05, ^**^ *p* < 0.01, and ^***^ *p* < 0.001 indicating significant differences and *n.s*. indicating no significance. G) The lysosome was consistent with that of the pro‐lysosomes, autolysosomes, and endolysosomes, all of which are indicated by Lamp1‐positive puncta. In the *SorL1 KO* hippocampus, both nuclear TFEB and autophagy markers were downregulated, indicating that pro‐lysosome and auto‐lysosome were reduced in the lysosome. In contrast, the number of Lamp1 positive puncta counts was increased in the *SorL1 KO* hippocampus, suggesting that endolysosomes were increased and accounted for most of the lysosomal flux when SorL1 was depleted.

Lysosomes indicated by Lamp1‐positive puncta include pro‐lysosomes, autolysosomes, and endolysosomes. Pro‐lysosomes primarily emerge from the TGN and eventually fuse with late endosomes or autophagosomes to degrade their inclusions.^[^ ^19a^ ^]^ To determine why the number of Lamp1‐positive puncta was increased (Figure 2G,I) but lysosomal digestion capacity was reduced in the *SorL1 KO* hippocampus (Figure 3D,E), we measured the abundance of lysosomal biogenesis factor transcription factor EB (TFEB) in subcellular fractions isolated from the hippocampus, as the nuclear entry of TFEB is necessary for lysosome biogenesis.^[^ ^37^ ^]^ The purity of the subcellular fractions was assayed with H3 in the nucleus and GAPDH in the cytoplasm (Supplementary S1E, Supporting Information). Although TFEB was upregulated ≈1.67‐fold in *SorL1 KO* hippocampal lysates, less TFEB was detected in the *SorL1 KO* nuclear fraction (only ≈40% of that in *WT* nuclei), while more TFEB appeared in the cytoplasm (approximately 1.34 times that in *WT*) (**Figure** **4**A,B). These results suggested that the increase in the number of Lamp1‐positive puncta in the *SorL1 KO* hippocampus may not have been due to enhanced lysosome biogenesis regulated by nuclear relocalization of TFEB.

The nuclear relocalization of TFEB is controlled by the phosphorylation of multiple upstream regulators, including serine/threonine‐protein kinase Akt, mitogen‐activated protein kinase kinase kinase kinase 3 (MAP4K3), glycogen synthase kinase 3 beta (GSK3β), extracellular signal‐regulated kinase 2 (ERK2), and mechanistic target of rapamycin complex 1 (mTORC1), at different sites.^[^ ^38^ ^]^ Among these regulators, the activation/inactivation of mTORC1 is closely correlated with lysosomes. Normally, activated mTORC1 localizes to lysosomes and phosphorylates TFEB to inhibit its entry into the nucleus.^[^ ^39^ ^]^ To determine whether TFEB entry into the nucleus is inhibited by mTORC1 activation, we measured the activity of mTORC1 upon phosphorylation of ribosomal protein S6 kinase 1 (S6K1) at the T389 site, which was 1.96‐fold greater in the *SorL1 KO* hippocampus than in the *WT* hippocampus (Figure 4C,D). An increase in S6K1^T389^ indicated that there was more activated mTORC1 in the *SorL1 KO* hippocampus, confirming that lysosome biogenesis was suppressed and that the presence of pro‐lysosomes was not responsible for the increased number of Lamp1‐positive puncta in the *SorL1 KO* rats.

The levels of the autophagy markers Beclin1, microtubule‐associated protein light chain 3 (LC3), and sequestosome 1 (p62/SQSTM1) were subsequently measured to explore whether the increase in the number of Lamp1‐positive puncta was due to an increase in the number of autolysosomes generated during autophagy enhancement in the *SorL1*‐deficient hippocampus. Beclin1 and LC3‐II/I were downregulated by 30% and 22.9%, respectively, compared to the levels in the *WT* hippocampus (Figure 4E,F), indicating that autophagy was perturbed in the *SorL1 KO* hippocampus, although p62 was unaffected (Supplementary S1F,G, Supporting Information). Based on these data, we concluded that the increase in the number of Lamp1‐positive puncta could be attributed to the presence of endolysosomes in the *SorL1 KO* hippocampus, leading to the obstruction of intracellular lysosomal digestion (Figure 4G).

### 2.3 Reprogrammed Energy Metabolism in the *SorL1*‐Null Hippocampus

**Figure 5:**
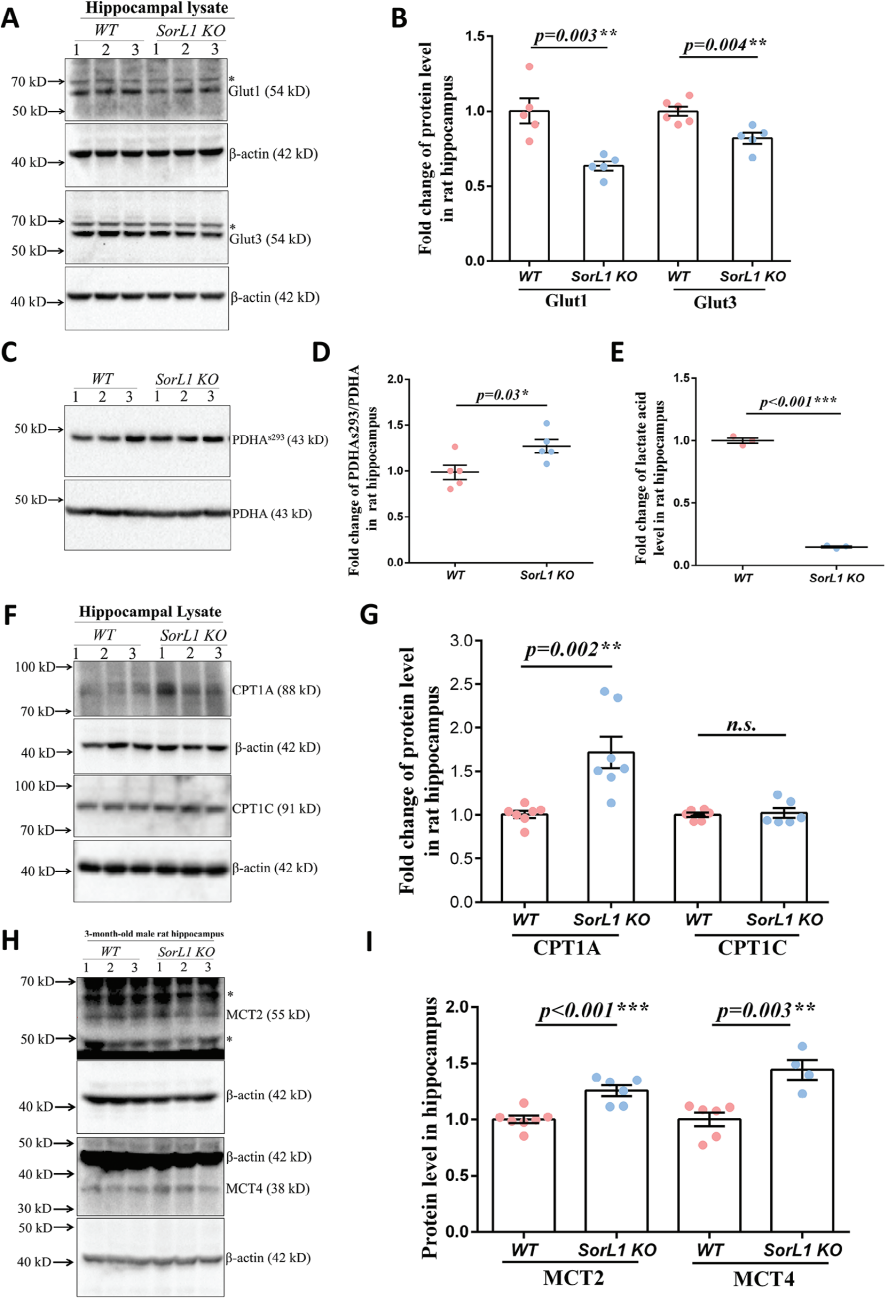
Fatty acid substitutes glucose to fuel the *SorL1*‐null hippocampus. A,B) Abundance of Glut1 and Glut3 in hippocampal lysates. The stray band is indicated by the asterisk. The protein levels of individual rats are presented by dots in B), and the bar chart shows the mean ± SEM values in B). Three independent experiments involving *WT* (*n* = 5) and *SorL1 KO* (*n* = 5) rats for Glut1 assay and *WT* (*n* = 6) and *SorL1 KO* (*n* = 5) rats for Glut3 assay were performed. C,D) The phosphorylation level of PDH^s293^ in the rat hippocampus was assayed. Data from individual rats are presented by dots in D), and the chart shows the mean ± SEM values. Three independent experiments with *WT* (*n* = 5) and *SorL1 KO* (*n* = 5) rats were performed. E) Tissue lactate levels measured in the rat hippocampus. Three independent experiments with *WT* (*n* = 3) and *SorL1 KO* (*n* = 3) rats were performed and the results are presented as the mean ± SEM. F,G) Protein levels of CPT1A and CPT1C were assayed in the rat hippocampus. The data from individual rats are presented as dots, and the bar chart shows the mean ± SEM values in G). Three independent experiments including *WT* (*n* = 7) and *SorL1 KO* (*n* = 7) rats for CPT1A assessment and *WT* (*n* = 6) and *SorL1 KO* (*n* = 6) rats for CPT1C measurement were performed. H,I) Protein levels of MCT2 and MCT4 in the rat hippocampus. The stray band is indicated by the asterisk. Data from individual rats are presented by dots, and the bar chart shows the mean ± SEM values in I). Three independent experiments included *WT* (*n* = 7) and *SorL1 KO* (*n* = 6) rats for MCT2 assessment. *WT* (*n* = 6) and *SorL1 KO* (*n* = 4) rats were used for MCT4 measurement. Three‐month‐old male rats were used for sample collection. Two‐tailed *t*‐tests were performed, with ^*^ *p* < 0.05, ^**^ *p* < 0.01, and ^***^ *p* < 0.001 indicating significant differences, and *n.s*. indicating no significant difference.

mTORC1 is a sensor of intracellular nutritional status and is active when adequate nutrition is supplied.^[^ ^40^ ^]^ In the adult brain, the primary carbon source is glucose.^[^ ^41^ ^]^ Generally, the capacity to transport glucose into the brain exceeds its requirement by two‐ to threefold,^[^ ^42^ ^]^ which is achieved by glucose transporter type1 (Glut1) and glucose transporter type 3 (Glut3), which are located at the plasma membranes of glia (astrocytes and oligodendrocytes) and neurons, respectively.^[^ ^43^ ^]^ We found that the abundances of Glut1 and Glut3 were decreased by ≈37% and 18%, respectively, in the *SorL1 KO* hippocampus (**Figure** **5**A,B), indicating reduced glucose requirements in the *SorL1 KO* rat brain. Glucose is oxidized to pyruvate through glycolysis and then decarboxylated through pyruvate dehydrogenase (PDH) in the mitochondria to generate acetyl‐CoA. We then measured PDH activity by assessing the phosphorylation level of the inhibitory site at S293 of PDH E_1_, namely, PDHA^s293^. It was upregulated ≈1.27‐fold in the *SorL1 KO* hippocampus (Figure 5C,D), indicating decreased PDH activity compared to that in the *WT* hippocampus. Furthermore, lactic acid levels were decreased by 85% in hippocampal tissue (Figure 5E) and by 32.8% in cardiac puncture blood in *SorL1 KO* rats (Supplementary S1H, Supporting Information). Decreased PDH activity and hippocampal lactate levels indicated that reduced pyruvate was generated from glucose and that aerobic glycolysis was attenuated in the *SorL1 KO* hippocampus.

Although glucose glycolysis was attenuated in the *SorL1 KO* hippocampus, increased mTORC1 activity indicated that there was no nutrient shortage (Figure 4C,D). Fatty acids are usually used as a substitute for fuel when glucose is lacking. They directly release acetyl‐CoA through β‐oxidation in the mitochondria, the process for which is independent of glycolysis. During fatty acid catabolism, fatty acyl‐CoA is activated in the cytoplasm and transported into the mitochondrial matrix by carnitine palmitoyl transferase (CPT), which is considered a rate‐limiting step of β‐oxidation.^[^ ^44^ ^]^ CPT1A and CPT1C are specifically expressed in astrocytes and neurons in the brain respectively.^[^ ^45^ ^]^ When *SorL1* was depleted, astrocytes‐specific CPT1A was upregulated 1.72‐fold, while neuron‐specific CPT1C appeared to be unaffected, suggesting that the rate‐limiting step of fatty acid β‐oxidation is specifically enhanced in astrocytes (Figure 5F,G), the type of cell that subcontracts the breakdown of fatty acids in the brain.^[^ ^46^ ^]^ Normally, in the brain, acetyl‐CoA forms ketone bodies in astrocytes, and is shuttled to neurons through monocarboxylate transporters (MCTs).^[^ ^47^ ^]^ MCT1 and MCT4 are specifically located in astrocytes, while MCT2 is expressed in neurons. We then measured the abundance of these proteins in hippocampal lysates and found that MCT2 and MCT4 were upregulated in *SorL1 KO* rats (Figure 5H,I), suggesting that these MCTs are involved in the shuttling of ketone bodies from astrocytes to neurons. However, MCT1 protein levels appeared to be decreased in the *SorL1 KO* hippocampus (Supplementary S1I,J, Supporting Information), indicating that this protein may be primarily responsible for lactate shuttling.

**Figure 6:**
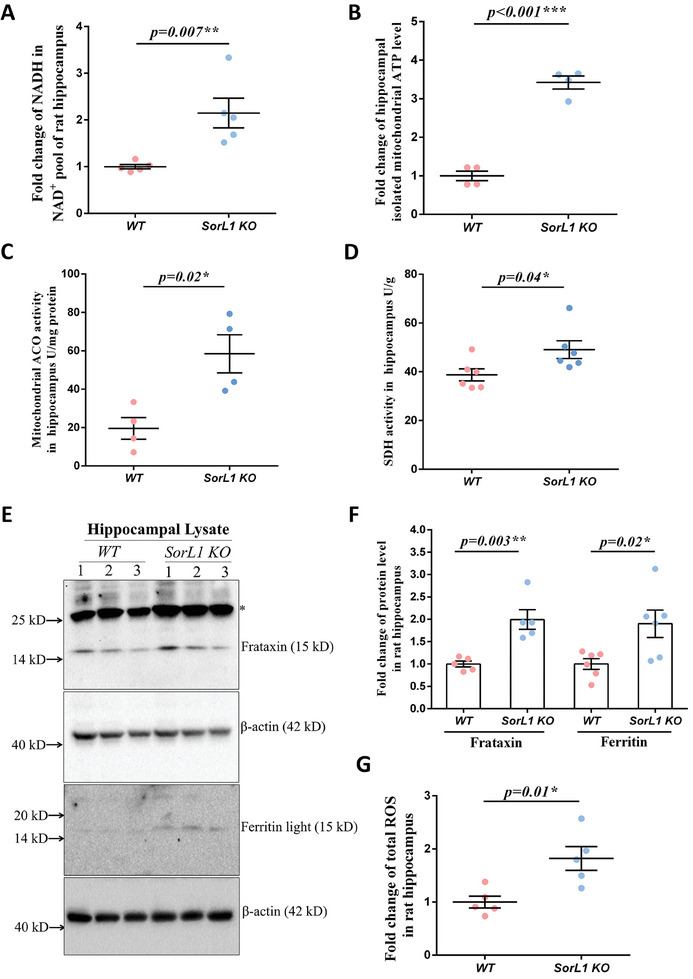
Iron enhances mitochondrial energetic metabolism in the *SorL1*‐null hippocampus. A) NADH levels were measured in hippocampal tissue. Data from individual rats are presented by dots as the mean ± SEM. Three independent experiments including *WT* (*n* = 5) and *SorL1 KO* (*n* = 5) rats were performed. B) ATP content assessment in isolated hippocampal mitochondria. Hippocampi were collected for mitochondrial isolation from 3‐month‐old male rats. Equal amounts of mitochondria were lysed for measurement. Data from individual rats are presented as dots, and the chart shows the mean ± SEM values. Three independent experiments including *WT* (*n* = 4) and *SorL1 KO* (*n* = 4) rats were performed. C) Mitochondrial ACO activity in the hippocampus was detected. Hippocampi were collected to isolate mitochondria. Equal amounts of mitochondria were lysed for the assessment. Three independent experiments with *WT* (*n* = 4) and *SorL1 KO* (*n* = 4) rats were performed. D) Measurement of SDH activity in the hippocampus. Hippocampi were collected and lysed for detection. Three independent experiments with *WT* (*n* = 6) and *SorL1 KO* (*n* = 6) rats were performed. E,F) Protein levels of frataxin and ferritin in the rat hippocampus were assayed. The stray band is indicated by the asterisk. Three independent experiments with *WT* (*n* = 5) and *SorL1 KO* (*n* = 5) rats were performed in the frataxin assay, and *WT* (*n* = 6) with *SorL1 KO* (*n* = 6) rats were performed in the ferritin assay. G) ROS measurement in hippocampal tissue. Total reactive oxygen species (ROS), including hydrogen peroxide, hydroxyl radical, singlet oxygen, nitric oxide, superoxide anion, peroxyl radical, and hydroperoxyl, were detected in the homogenate of fresh rat brain hippocampal tissue. Three independent experiments with *WT* (*n* = 5) and *SorL1 KO* (*n* = 5) rats were performed. Three‐month‐old male rats were used for sample collection. Two‐tailed *t*‐tests were performed with ^*^ *p* < 0.05, ^**^ *p* < 0.01 and ^***^ *p* < 0.001 indicating significant differences.

The oxidation of acetyl‐CoA releases high‐energy electrons during the tricarboxylic acid cycle (TCA cycle), and these electrons are captured by nicotinamide adenine dinucleotide (NAD^+^) to generate NADH, which is the electron donor for the electron transport chain (ETC) that drives ATP synthases. The level of NADH in the hippocampal NAD^+^ pool was found to be 2.15 times greater in the *SorL1 KO* hippocampus than in the *WT* hippocampus (the proportion of NADH in the NAD^+^ pool was maintained at ≈27.9%) (**Figure** **6**A). Moreover, mitochondria were isolated from the hippocampus to determine the ATP level in the matrix, which was 3.42 times greater in *SorL1 KO* rats than in *WT* rats (Figure 6B). Both the inlet substrate NADH and the outlet product ATP of the respiratory chain were significantly enriched in the *SorL1 KO* hippocampus, suggesting that oxidative phosphorylation (OXPHOS) was enhanced through fatty acid oxidation but was not dependent on glucose glycolysis.

In the *SorL1 KO* hippocampus, glucose glycolysis was attenuated, but the ATP supply was elevated through an increase in the TCA cycle and OXPHOS, indicating reprogrammed metabolism in the astrocytes of the *SorL1 KO* hippocampus, in which fatty acids, rather than glucose, were used as the primary carbon source in young adult rats.

### 2.4 Lysosome‐Mitochondrial Crosstalk Through Iron in the *SorL1*‐Null Hippocampus

In the mitochondrial matrix, the TCA cycle is regulated by enzymes that are sensitive to calcium concentration, including PDH at the s293 site.^[^ ^48^ ^]^ The increased phosphorylation of PDHA^s293^ indicated that the increase in NADH generated through the TCA cycle in the *SorL1 KO* hippocampus may not be due to the increase in mitochondrial matrix calcium (Figure 5C,D). Our previous study in the hippocampus of *APP KO* rats indicated that full‐length APP prohibited calcium flow from the ER lumen to the mitochondrial matrix on mitochondria‐associated membranes (MAMs).^[^ ^49^ ^]^ The up‐regulation of full‐length APP in the hippocampus of *SorL1 KO* rats may also increase MAM‐localization to the ER and inhibit mitochondrial calcium influx from the ER, which is consistent with the conclusion that more inhibitory phosphorylation of PDHA and decreased mitochondrial matrix calcium levels in the *SorL1 KO* rat hippocampus.

It was previously shown that transcriptomic changes in iron homeostasis‐related genes occur in *SorL1*‐null mutant zebrafish,^[^ ^50^ ^]^ and we found that the ferritin content in the hippocampus of *SorL1 KO* rats was greater than that in the hippocampus of *WT* rats (Figure 3B). These findings prompted us to investigate whether iron promotes the TCA cycle in the mitochondrial matrix of the *SorL1 KO* hippocampus. The activities of aconitase (ACO) and succinate dehydrogenase (SDH), two enzymes in the TCA cycle that are specifically regulated by the mitochondrial matrix iron^[^ ^51^ ^]^ were increased 2.99‐fold and 1.27‐fold, respectively, in the mitochondria of the *SorL1 KO* hippocampus (Figure 6C,D). These results demonstrated that increased mitochondrial matrix iron upregulated the TCA cycle through ACO and SDH in the *SorL1 KO* hippocampus.

Mitochondrial imported iron is utilized in three metabolic pathways, heme‐synthesis, Fe‐S cluster biogenesis, and the storage of mitochondrial ferritin.^[^ ^36^ ^]^ Fe‐S clusters are essential components of mitochondrial ETC complexes.^[^ ^52^ ^]^ SDH is also an integral component of the mitochondrial ETC complex II, and its activation suggests that increased OXPHOS requires more respiratory chain complexes, which allows some of the mitochondrial iron to be used for Fe‐S cluster synthesis. The biosynthesis of the Fe‐S cluster involves a group of dedicated multimeric protein complexes, with frataxin as part of the core complex in the mitochondria.^[^ ^53^ ^]^ We measured the protein level of frataxin in the rat hippocampus, and the level in the *SorL1 KO* hippocampus was 1.99‐fold greater than that in the *WT* hippocampus (Figure 6E,F). Excess iron is bound by ferritin to prevent the generation of oxygen radicals;^[^ ^54^ ^]^ thus, the protein level of ferritin was examined in hippocampal lysates and appeared to be 1.9 times greater in the *SorL1 KO* hippocampus than in the *WT* hippocampus (Figure 6E,F). Even so, the reactive oxygen species (ROS) level was increased by ≈1.82‐fold in the *SorL1 KO* hippocampus (Figure 6G).

Mitochondrial iron levels are linked to changes in mitochondrial dynamics, with the loss of mitofusin 2 protein (MFN2) associated with mitochondrial iron overload,^[^ ^55^ ^]^ mitochondrial fusion protein optic atrophy 1 (OPA1) cleavage associated with cellular iron overload,^[^ ^56^ ^]^ and cellular iron overload associated with dynamin‐related protein 1 (DRP1) (Ser637) dephosphorylation.^[^ ^57^ ^]^ We then measured the levels of these proteins in hippocampal lysates (Supplementary S2A,B, Supporting Information) and found that the levels of the short forms of OPA1 and Drp1 were significantly decreased in the *SorL1 KO* hippocampus. Consistently, the mitochondrial morphology was observed and more rod‐shaped mitochondria were found in the neuronal soma of *SorL1 KO* hippocampus (Supplementary S2C,D, Supporting Information). These data suggested that the homeostasis of mitochondria is altered in response to increased mitochondrial respiratory and metabolic demands, which are regulated by iron, an intracellular second messenger. However, this disturbance may not ripple the ER, as the ER stress‐specific indicator Grp78 did not significantly differ between the *SorL1 KO* and *WT* hippocampi (Supplementary S2E,F, Supporting Information).

The iron absorbed by mitochondria is derived mainly from the cytoplasm, namely, the labile iron pool (LIP), and the lysosome, which is the iron source of the LIP for the uptake and release of iron.^[^ ^17^ ^]^ Disorder of the ELN caused by *SorL1* deficiency leads to excess intracellular iron, which, as an intracellular second messenger, enhances hippocampal mitochondrial respiration and maintains a high energy charge. At this point, the cells are not deficient in carbon sources because of increased fatty acid oxidation, so mTORC1 is more active in the *SorL1 KO* hippocampus, and less glucose is needed.

### 2.5 Disrupted Spatial Memory in *SorL1*‐Null Young Rats

**Figure 7:**
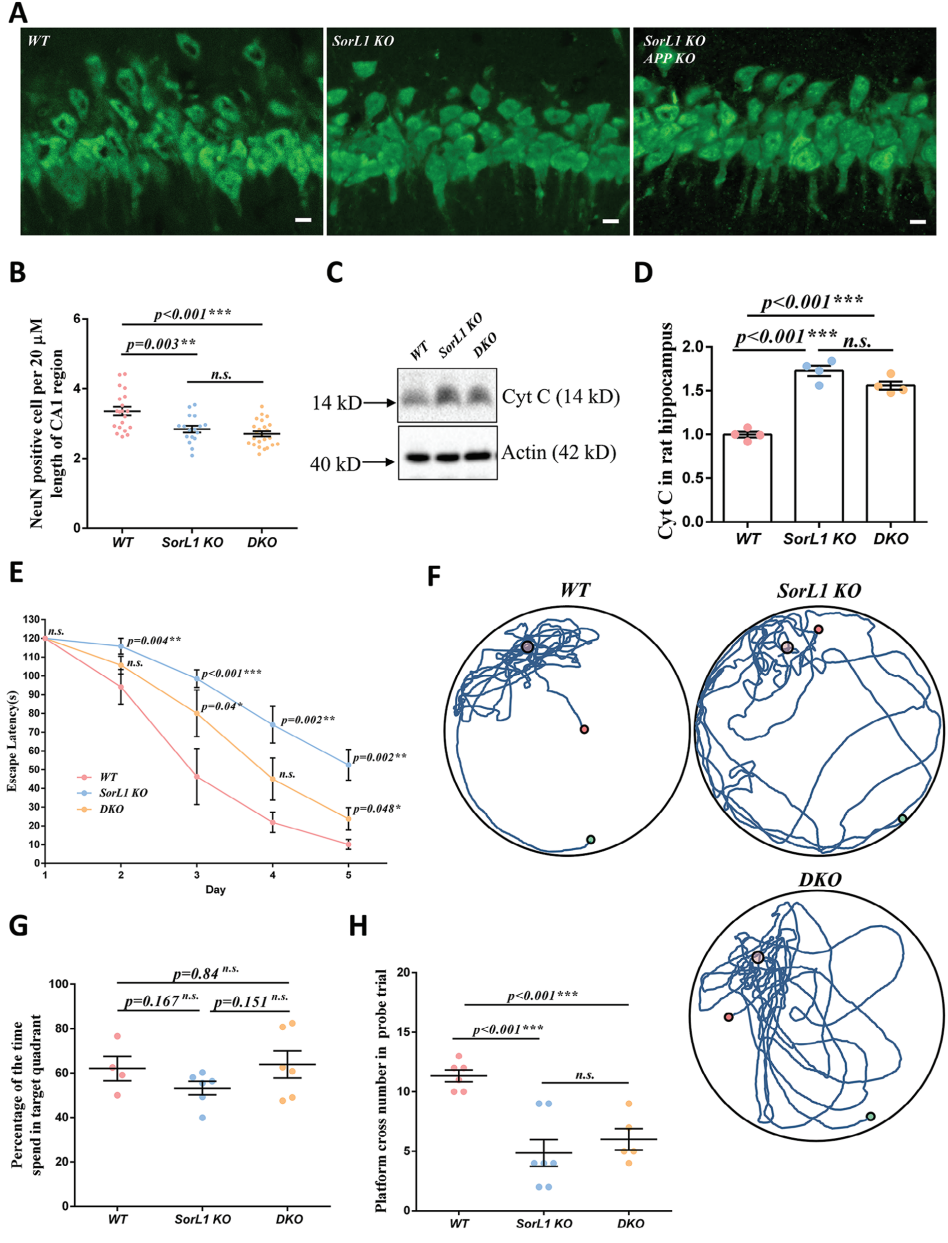
Hippocampal neurodegeneration in *SorL1*‐null rats. A,B) Immunostaining of rat brain slices with a NeuN antibody. Coronal slices from each rat were prepared to visualize the bilateral hippocampus. Two slices per rat were observed under a 60× objective, as shown in A). Scale bar = 10 µm. Neurons in the hippocampal CA1 region were photographed and counted. The average number of neurons 20 µm in length in the CA1 region from each image is indicated by a dot. Data from each genotype are presented as the mean ± SEM in B). Three independent experiments with *WT* (*n* = 6), *SorL1 KO* (*n* = 6), and *DKO* (*n* = 6) rats were performed. C,D) Fold change of Cyt C was assayed in the rat hippocampus. Three independent experiments with *WT* (*n* = 4), *SorL1 KO* (*n* = 4), and *DKO* (*n* = 4) rats were performed in the assay. Data from individual rats are presented as dots, and the chart shows the mean ± SEM values in D). E–H) MWM test with 3‐month‐old male rats. Three independent experiments including at least four rats per genotype were performed. The escape latency during the training is shown in E). Representative tracks of individual rats in the probe trail are shown in F). The percentage of the time spent in the target quadrant in the probe trial is shown in G). The number of platform crossings in the probe is shown in H). Data from individual rats are indicated by dots, and the mean ± SEM for each genotype is presented in G) and H). Three‐month‐old male rats were used for sample collection. One‐way ANOVA was performed, ^*^ *p* < 0.05, ^**^ *p* < 0.01, and ^***^ *p* < 0.001 indicate significant differences, and *n.s*. indicates no significance.

To further investigate the effects of ELN disorder on hippocampus‐dependent spatial memory in *SorL1 KO* rats, we observed the lamellar structure of neurons in the CA1 region through anti‐NeuN antibody staining. The pyramidal neurons in the CA1 region of *WT* rats were arranged in layers, while local vacancies appeared in the *SorL1 KO* hippocampus and the CA1 region of *DKO* rats exhibited a pathology similar to that of *SorL1 KO* rats (**Figure** **7**A). The number of NeuN‐positive neurons was calculated to indicate morphological alterations in the hippocampus, which were significantly reduced in *SorL1 KO* rats (≈3.4 neurons per 20 µm of the CA1 region in *WT* rats but 2.8 neurons in *SorL1 KO* rats). The number of neurons in the CA1 region in *DKO* rats was comparable to that in *SorL1 KO* rats, with no significant difference (Figure 7B). To further determine whether increased ROS causes apoptosis in the hippocampi of gene‐deficient rats, we detected the protein levels of apoptosis marker cytochrome c (Cyt C) in the hippocampal lysate. In the *SorL1 KO* and *DKO* rats, Cyt C was significantly upregulated compared to that in the *WT* rats, suggesting that apoptosis was increased in the hippocampus due to genetic defects (Figure 7C,D).

The Morris water maze (MWM) test was used to assess hippocampus‐dependent spatial memory in the rats (Figure 7E–H). After 5 days of training, the *WT* rats learned to find the escape platform, and their time spent gradually decreased. After training on the second day, the performance gap between *SorL1 KO* rats and *WT* rats gradually widened, and the performance of *DKO* rats was between that of *WT* and *SorL1 KO* rats (Figure 7E). In the probe test, the cumulative distance and swimming speed of the genetically defective rats were comparable to those of the *WT* rats (Supplementary S3A,B, Supporting Information), suggesting that motor ability was undisrupted by gene deficiency in these young rats. The time spent in the target quadrant by the *WT* rats was >60% of the total 2 min, and the average number of platform crossings was 11.3 (Figure 7F–H), indicating the normal learning and memory ability of the *WT* rats. *SorL1 KO* rats stayed in the target quadrant for 53.3% of the total time and crossed the platform position ≈5 times. Compared to *WT* rats, *SorL1 KO* rats were confused about the exact position of the escape platform, suggesting mild spatial memory impairment. The MWM performance of the *DKO* rats was slightly better than that of the *SorL1 KO* rats, as the *DKO* rats crossed the platform 6 times and stayed in the target quadrant for 63.9% of the total time, but these differences were not significant (Figure 7F–H). These results indicated that *APP* depletion could not completely rescue the spatial memory impairment caused by *SorL1* deficiency, which was consistent with the previously observed changes in ELN morphology in the hippocampal neurons of *DKO* rats. Hippocampal degeneration and spatial memory impairment in *SorL1*‐deficient young rats suggested that *SorL1* is necessary for maintaining hippocampal physiology, which is not completely mediated by APP in the early pathology of AD.

## 3 Discussion

Based on our research, we believe that the pathological function of SorL1 in neuronal degeneration involves the regulation of the global ELN, which is critical for the sensing, absorption, and distribution of nutrients and the digestion, degradation, and reuse of metabolites and damaged organelles. When *SorL1* is absent, the hippocampus gives up glucose as fuel and rebuilds material‐energy‐structure homeostasis based on fatty acid oxidation. To achieve this, an intracellular membrane system is required for the rebuilding process. In fact, sorting of APP and Aβ on endosomes by SorL1 has demonstrated the ability of SorL1 to control membrane structure. As shown in Figure 1I, in the retrograde sorting of APP by SorL1 from the early endosome to the TGN, the endosomal membrane needs to bud toward the cytoplasmic side, and with the help of anchored microtubules, the budding vesicles are drawn into a rod shape until they are separated from the endosomal vesicles.^[^ ^58^ ^]^ However, endosomal Aβ sorting to the lysosome requires budding toward the lumen side through invagination of the endosomal membrane, namely, endosome maturation or multivesicular body (MVB) formation.^[^ ^58^ ^]^ Afterward, mature endosomes fuse with lysosomes to form endolysosomes, and the endosomal content is degraded by lysosomal digestive enzymes. Therefore, we suggest that SorL1 may act as a regulator to recruit various factors leading the endosomal membrane bending toward to cytoplasm and lumen side. When SorL1 is absent, the endosomal membrane cannot bend properly to affect vesiculation or indention, resulting in endosomal swelling, which affects the material sorting and degradation mediated by the global ELN, as well as lysosomal pH and its regulation of digestion, cholesterol, and iron homeostasis. To assess whether lysosomal pH is affected, we detected the lysosomal pH regulator lysosomal transmembrane protein 175 (TMEM175)^[^ ^59^ ^]^ in *SorL1 KO* hippocampi, which was significantly upregulated (Supplementary S3C,D, Supporting Information), suggesting an abnormal lysosomal pH due to SorL1 deficiency.

These disturbances disrupt the homeostasis of the original material‐energy‐structure through glucose catabolism and reprogram energy metabolism based on fatty acids in the *SorL1 KO* hippocampus. The transcriptional co‐activator PPAR‐gamma co‐activator‐1 al (PGC1α), a downstream transcription factor of TFEB and its target genes, has been proven to be involved in fatty acid oxidation^[^ ^60^ ^]^ and should mediate enhanced fatty acid catabolism in the *SorL1 KO* hippocampus, as its abundance increased approximately 1.54‐fold (Supplementary S3E,F, Supporting Information). Furthermore, we also examined the levels of NF‐E2‐related factor 2 (NRF2) and mitochondrial transcription factor A (TFAM), two transcription factors that are downstream of PGC1α and regulate mitochondrial biogenesis. Decreased TFAM levels in the *SorL1 KO* hippocampus indicated that mitochondrial biogenesis was inhibited, likely because of the high intracellular energy charge.

Interestingly, a disrupted ELN results in altered energy metabolic homeostasis in the *SorL1*‐deficient hippocampus. We suggest that the relationship between ELN disturbances and changes in energy metabolism is caused by changes in iron homeostasis regulated by ELN. Iron is absorbed by the endosome through the endocytosis pathway and is eventually transported to the lysosome. In the acidic lumen of late endosomes and lysosomes, Fe^3+^ is reduced to Fe^2+^, which is then released by the lysosomes into the cytoplasmic LIP for further use. Mitochondria take up iron into their matrix and use it for heme synthesis, Fe‐S cluster biogenesis, or storage with mitochondrial ferritin.^[^ ^36^ ^]^ Neuronal ELN destruction and lysosomal dysfunction, accompanied by iron accumulation were observed in the *SorL1*‐deficient hippocampus, suggesting that the disorder of ELN caused by *SorL1*‐deficiency leads to intracellular iron accumulation. According to our data, excessive intracellular iron enters the mitochondrial matrix and drives the TCA cycle by activating iron‐dependent ACO and SDH, thereby increasing ATP productivity, Fe‐S cluster synthesis, and mitochondrial iron storage and promoting energy metabolism, and the carbon source consumed is fatty acids. It is not clear whether iron deposition in neurons is a key factor in inhibiting glucose metabolic capacity and promoting fatty acid decomposition. Recent reports have shown that increased glucose intake introduces a series of downstream metabolic changes, including pyruvate oxidation and TCA cycle enhancement, stimulating fatty acid synthesis, and ultimately promoting ferroptosis,^[^ ^61^ ^]^ suggesting a link between iron homeostasis and glucose‐lipid metabolic reprogramming. Similarly, there should be an intrinsic mechanism underlying the relationship between increased energy homeostasis and hippocampal degeneration. We speculated that elevated ROS levels might be the main cause of neuron degeneration in the *SorL1*‐deficient hippocampus. We believe that ROS results from the Fenton reaction caused by iron deposition in the cell, or the increase in oxidative phosphorylation leading to more electrons escaping from the mitochondrial respiratory chain, or both. The following questions need to be further answered: 1) Is iron deposition a direct factor that promotes fatty acid oxidation in *SorL1* defective metabolic reprogramming? 2) Do ROS originate from the respiratory chain or iron accumulation? 3) Are ROS the main cause of hippocampal degeneration in *SorL1*‐deficient rats? To solve these problems, it is necessary to inhibit corresponding targets, such as neutralizing excessive iron ions by using deferoxamine, to observe whether fatty acid oxidation and energy metabolism are weakened and whether ROS are reduced, which is conducive to improving energy metabolic homeostasis and preventing degeneration of the hippocampus.

We also hypothesized that starvation improved metabolic reprogramming caused by *SorL1* deficiency. During starvation, the glycogen of *WT* rats is exhausted, and the fatty acid mobilization is carried out to generate ketones in the liver. These ketones are released into the blood at concentrations up to millimoles, directly crossing the blood‐brain barrier to power the brain. In *SorL1*‐deficient rats, brain tissue already relies on fatty acid oxidation rather than glucose for energy without starvation. After starvation treatment, blood ketones enter the brain to supply energy, and the requirement for brain tissue to oxidize its own fatty acids should be reduced.

The commonly accepted pathology of AD involving SorL1 is that SorL1 mediates retrograde transport of APP from the endosome to the TGN, a process that inhibits APP processing into amyloid plaques.^[^ ^13^, ^33^ ^]^ Recent studies have shown that SorL1 deficiency causes early endosomes swelling in human induced pluripotent stem cell‐derived neurons (hiPSC‐Ns)^[^ ^13^ ^]^ and minipig cortical neurons.^[^ ^14^ ^]^ However, whether the endosome enlargement of *SorL1*‐deficient hiPSC‐Ns depends solely on the processing of APP into amyloid plaques in the endosome, especially in individuals, is unclear. At the beginning of the study, we expected to observe that APP depletion completely rescued *SorL1*‐null induced ELN abnormalities and hippocampal degeneration in *DKO* rats, which should prove that APP accumulation is the only cause of these pathological changes in SorL1 deficiency. However, our findings do not support this speculation. Therefore, we demonstrated that the regulatory role of SorL1 in the ELN and lysosomes is not merely dependent on APP; thus, we studied the pathology of SorL1 deficiency in the hippocampus. In this study, we demonstrated that *SorL1* deficiency disrupts the ELN, thereby affecting lysosomal‐mediated intracellular iron homeostasis and leading to cytoplasmic iron accumulation. More cytoplasmic iron enters the mitochondrial matrix to activate iron‐dependent enzymes and drive the TCA cycle. Moreover, more mitochondrial iron is used for the synthesis of Fe‐S clusters, which are necessary for the electron transport chain to generate ATP. Therefore, the entry of iron into the mitochondrial matrix increases the cell's energy metabolism, which necessarily consumes more carbon sources, normally glucose as the primary carbon source for brain tissue. However, in the hippocampus of *SorL1‐*deficient rats, we unexpectedly found that fatty acids replaced glucose to accommodate enhanced energy metabolism. Excessive iron accumulation might cause an intracellular Fenton reaction, and/or more escaping electrons from the mitochondrial respiratory chain due to enhanced oxidative phosphorylation and increased ROS levels, which may be the main cause of hippocampal degeneration in *SorL1*‐deficient rat brains.

We established a new and reliable research model to study the pathological mechanism of *SorL1* defects, and the causal relationships among various pathological factors in this model need to be further explored. AD is thought to be a group of heterogeneous diseases, and its causes are varied. Our research elucidates the metabolic‐ and cytopathology of *SorL1* deficiency in the early stage of AD. In addition, because many of the pathological manifestations of *SorL1*‐null rats are similar to AD pathology, we believe that this rat line can be used as a new model for preclinical AD drug development.

## 4 Experimental Section

### Generation of Gene Knockout Rats by TALEN

TALEN was designed to target exon 2 in the *SorL1* gene of Sprague‐Dawley (SD) rats, and the chimera rats were generated by K&D Gene Technology (Wuhan, China). The TALEN mRNA was transcribed in vitro and injected into fertilized eggs of rats. Targeted embryos were transferred into the fallopian tubes of the surrogate mother. The F0 generation was genotyped through PCR using the forward primer 5′‐TGCCCTGACCCCACACCT‐3′ and reverse primer 5′‐ACCTGTGAGATTTGCAGCCAC−3′. Chimaeras with *SorL1* gene mutations were collected and hybridized with *wild‐type* SD rats for at least five generations to acquire heterozygotes. *SorL1*‐null rats were produced through heterozygotes crossing.

### Rat Husbandry and Treatment

The animal husbandry and treatment procedures were approved by the Institutional Animal Care and Use Committee. Rats were housed in the Animal Biosafety Level 3 Laboratory of Wuhan University. All rats were fed a standard diet and allowed to drink freely. The housing room was kept at an ambient temperature of 20 °C, with a humidity of 58% and fresh air. The fixed housing density was three rats per cage. All rats were exposed to a regular light/dark rhythm with 12 h per phase, switching at a fixed time of day.

Male rats aged 3 months were used for sample collection unless otherwise specified. Dissection was performed immediately after decapitation for fresh tissue samples. For fixed brain collection, rats were intraperitoneally injection with pentobarbital (2% in w/v) at a dose of 0.5 mL/0.1 kg of rat body weight.

### Antibodies and Reagents

The following antibodies were used for immunoblotting: SorL1(A13047, Proteintech, 1:1000), APP(D260097‐0200, BBI, 1:1000), β‐actin(AC026, ABclonal, 1:2000), EEA1 (A0592, ABclonal, 1:1000), Lamp1 (A16894, ABclonal, 1:1000), CTSB(12216‐1‐AP, Proteintech, 1:1000), CTSD(21327‐1‐AP, Proteintech, 1:2500), TFEB (13372‐1‐AP, Proteintech, 1:1000), Histone 3 (A2348, ABclonal, 1:1000), Gapdh (AF0006, Beyotime, 1:1000), S6K1‐T389 (AP0564, ABclonal, 1:1000), S6K1 (A16658, ABclonal, 1:1000), Beclin 1(11306‐1‐AP, Proteintech, 1:1000), LC3 (14600‐1‐AP, Proteintech, 1:1000), GLUT1 (21829‐1‐AP, Proteintech, 1:1000), GLUT3 (20403‐1‐AP, Proteintech, 1:1000), PDHA^S293^ (AP1022, ABclonal, 1:1000), PDHA (A1895, ABclonal, 1:1000), CPT1A (A5307, ABclonal, 1:1000), CPT1C (A13849, ABclonal, 1:1000), MCT2(20355‐1‐AP, Proteintech, 1:1000), MCT4(22787‐1‐AP, Proteintech, 1:1000), Frataxin (A11785, ABclonal, 1:1000), Ferritin(10727‐1‐AP, Proteintech, 1:1000), P62(A19700, ABclonal, 1:1000), MFN2 (12186‐1‐AP, Proteintech, 1:1000), OPA1 (ab42364, Abcam, 1:1000), Drp1 (ab56788, Abcam, 1:1000), Grp78 (11587‐1‐AP, Proteintech, 1:1000), PGC1α(66369‐1‐IG, Proteintech, 1:1000), NRF2(16396‐1‐AP, Proteintech, 1:1000), TFAM(A13552, ABclonal, 1:1000), MCT1(20139‐1‐AP, Proteintech, 1:1000), Cyt C (A4912, ABclonal,1:1000), TMEM175 (19925‐1‐AP, Proteintech, 1:1000).

The following antibody was used for immunofluorescence: NeuN (MAB377, Millipore, 1:800), EEA1 (A0592, ABclonal, 1:1000), Lamp1 (A16894, ABclonal 1:100), SorL1(22592‐1‐AP, Proteintech, 1:100).

### Tissue Collection

For fresh tissue collection, the hippocampus was quickly separated after decapitation. Unless otherwise specified, subsequent operations were performed in an ice bath. The tissue was cut into pieces, and homogenized with a grinding rod for 20 strokes and then in a Teflon glass homogenizer at 1 800 rpm for another 20 strokes. The homogenate was quickly used for further analysis.

For fixed brain collection, deeply anesthetized rats were intracardially perfused with 0.1 m PBS and 4% paraformaldehyde for brain fixation. Afterward, the brain was dissected and fixed in paraformaldehyde at 4 °C overnight and dehydrated in 10%, 20%, and 30% sucrose in PBS (w/v) successively. Frozen sections (14 µm) were obtained and stored at −80 °C.

### Immunofluorescence Assay

Hippocampal sections were washed with PBS for 15 min at RT and blocked with 5% goat serum and 2% Triton‐X/PBS for 1.5 h at RT. Primary antibodies were incubated overnight at 4 °C. After washing with PBS at RT for 30 min, the dye‐labeled secondary antibodies were stained at RT for 2 h. All the incubation steps were performed in a wet box. Sections were then mounted in a DAPI (VECTASHIELD) medium and observed with a Nikon A1 confocal microscope under a 60× objective. The images were analyzed with ImageJ. The consistent parameters of each image were ensured for the quantification assay.

### Subcellular Fractionation in the Hippocampus

Lysosome fractionation was isolated on ice with a lysosome extraction kit (BB‐3603, Bestbio, Shanghai) according to the manufacturer's instructions. Hippocampal tissues were dissected and cut into pieces in the extraction buffer at a w/v ratio of 1:100. After incubating the mixture on ice for 10 min, the tissues were ground with a grinding rod for 20 stokes and then immediately homogenized in a Teflon glass homogenizer at 1 800 rpm for 20 strokes. The lysate was centrifuged at 1000 × g for 5 min at 4 °C to acquire the supernatant. The obtained supernatant was centrifuged at 3000 × g and 5000 × g, each for 10 min, to collect the supernatant. This supernatant was centrifuged at 18000 × g for 20 min, and the obtained precipitate was added to lysosomal extraction buffer and centrifuged at 18 000 × g for 20 min. The final collected precipitate was the lysosomes fraction, and it was lysed for subsequent detection.

Nuclear and cytoplasmic isolation was performed on ice with an extraction kit (BB‐36022, Bestbio, Shanghai) according to the manufacturer's instructions. The hippocampi were dissected and cut into pieces in extraction buffer at a w/v ratio of 1:100. The lysate was shaken for 30 min and centrifuged at 1200 × g for 5 min at 4 °C. The supernatant was the cytoplasmic fraction. The pellet was washed in cold PBS and centrifuged at 2000 × g for 15 min at 4 °C, which was the nuclear fraction.

### Cholesterol and Iron Content Assay in the Hippocampus

Total cholesterol and free cholesterol in the hippocampal tissue were measured with assay kits according to the manufacturer's instructions. The hippocampus was collected and weighed as soon as possible. Tissue samples were homogenized in lysis buffer at a tissue mass (g) to extract solution volume(mL) ratio of 1:10 on ice and centrifuged at 10 000 × g at 4 °C for 10 min. The liquid supernatant was assessed with a total cholesterol assay kit (AKFA002M, BoxBio, Beijing) and a free cholesterol assay kit (AKFA001M, BoxBio, Beijing).

For tissue iron assessment, the hippocampus was homogenized in extraction solution at a tissue mass (g): volume of extract solution (mL) ratio of 1:10, and the homogenate was then assayed with crude mitochondrial samples obtained from the rat hippocampus using a tissue iron content assay kit (AKIC001M, BoxBio, Beijing) according to the manufacturer's instructions.

### TEM

Hippocampi were dissected and fixed in cold 2.5% glutaraldehyde overnight. The tissue was then postfixed in 1% osmium tetroxide and contrasted with 1% uranyl acetate, dehydrated in increasing ethanol concentrations, and embedded in Durcupan. Tissue blocks were then sliced into ultrathin sections. The sections were observed with a transmission electron microscope (HT7700, Hitachi).

### Lactate Assay in the Serum and Hippocampus

A cardiac puncture was performed after rat decapitation for serum collection. The hippocampus was homogenized in normal saline solution at a weight (g) to volume (mL) ratio of 1:4.5 for the lactate assay. The lactate assay was performed with a lactate assay kit (A019‐2‐1, Jiancheng, Nanjing) according to the manufacturer's instructions.

### Hippocampal NADH, ROS Content, and SDH Activity Assays

Hippocampal tissues were dissected and lysed in extraction buffer at a w/v ratio of 1:100. For the NADH assay, the homogenate was centrifuged at 12 000 × g for 10 min at 4 °C. The supernatant was collected, and the NADH and NAD^+^ levels were measured with an NAD^+^/NADH assay kit (S0175, Beyotime, Shanghai) according to the manufacturer's instructions. For the ROS assay, the homogenate was centrifuged at 1000 × g for 10 min at 4 °C. The supernatant was collected and incubated with an O13 probe (BB‐470512, Bestbio, Shanghai) at 37 °C for 30 min. Hippocampal ROS were measured under a 488 nm excitation laser in a fluorescence microplate reader according to the manufacturer's instructions. For the SDH assay, the homogenate was centrifuged at 11 000 × g for 10 min at 4 °C. The supernatant was measured with an SDH activity assay kit (AKAC011M, BoxBio, Beijing) according to the manufacturer's instructions.

### ATP Content and ACO Activity Measurement in Hippocampal Mitochondria

Hippocampal tissues were dissected and lysed in extraction buffer at a w/v ratio of 1:100. The homogenate was centrifuged at 11 000 × g for 15 min at 4 °C. The pellet was the mitochondrial fraction, which was collected and washed in the extraction buffer once. The mitochondrial fraction was lysed in lysis buffer and ultrasonicated (300w, 3s on, 9s off for 15 cycles) on ice. The mixture was centrifuged at 5000 × g for 2 min at 4 °C, and the supernatant was collected for the ATP content assay (S0027, Beyotime, Shanghai) and ACO activity assay (AKAC008M, BoxBio, Beijing) according to the manufacturer's instructions.

### MWM Test

Six male rats of each genotype at similar weights were collected for the experiment. The rats were made to interact with the experimenter for a week at a fixed time and place to acclimate them to the smell and touch. In accordance with the MWM protocol,^[^ ^62^ ^]^ the rats were subjected to a 5‐day place navigation experiment in which they were placed in four trials a day facing the wall of a pool from four entry points, and the time to find the escape platform was recorded. On the sixth day, the spatial probe experiment was conducted by removing the escape platform and then placing the rats into the pool at a fixed entry point to record their swimming trajectory within a certain period to examine their memory of the original platform. The experiment was filmed, and the video was analyzed by Smart 3.0 software (Panlab).

### Statistical Analysis

All data were statistically analyzed using GraphPad Prism 7.0. Data for each assay were obtained from at least three independent experiments unless otherwise stated. The data are expressed as the mean ± SEM and were analyzed using Student's *t*‐test and one‐way ANOVA as indicated. Significant differences are indicated, with asterisks with 0.01 ≤ ^*^ *p* < 0.05, ^**^ *p* < 0.01, and ^***^ *p* < 0.001 indicating significant differences, and *n.s*. indicating no significant difference.

### Ethics

All procedures involving animals were approved by the Animal Ethics Committee of Wuhan University. All animal studies were performed according to protocols approved by the Wuhan University Animal Committee (WP20230235). All members participating in this study received training in ethics and animal handling from Wuhan University.

## Conflict of Interest

The authors declare no conflict of interest.

## Author Contributions

Y.Y. and Y.C. contributed equally to this work. Y.W., L.W., and S.C. conceived the study. L.W. and S.C. supervised the project. Y.W., Y.Y., Y.C., L.W., and S.C. contributed to writing the manuscript. Y.W., Y.Y., Y.C., A.A., C.H., L.W., and S.C. analyzed and interpreted the data; Y.W., Y.Y., Y.C, A.A., Y.W., C.H., and Z.M. conducted experiments and acquired data; Y. W., Y.Y., Y.C., A.A., C.H., Z.M., Y.S, M.Z., Y.H., C.X., X.C., Z.L., and J.C. involved in methodology.

## Supporting information

Supporting Information

Supporting Information

## Data Availability

The data that support the findings of this study are available in the supplementary material of this article.
