# Supplementary material for: Endo‐Lysosomal Network Disorder Reprograms Energy Metabolism in SorL1‐Null Rat Hippocampus
Source: Adv Sci (Weinh). 2024 Sep 3;11(41):2407709. doi: 10.1002/advs.202407709 (PMC11538633; doi:10.1002/advs.202407709)
Supplement: Supplementary file 1 — Supporting Information [file ADVS-11-2407709-s002.docx]

**Supplementary material for:**

**Endo-lysosomal network disorder reprograms energy metabolism in the *SorL1*-null rat hippocampus**

Yajie Wang^1^, Yuting Yang^1†^, Ying Cai^1†^, Ayikaimaier Aobulikasimu^1^, Yuexin Wang^1^，Chuanwei Hu^1^, Zhikang Miao^1^, Yue Shao^1^, Mengna Zhao^1^, Yue Hu^1^, Chang Xu^1^, Xinjun Chen^1^, Zhiqiang Li^1^, Jincao Chen^1^, Lianrong Wang^1,2^*, Shi Chen^1,3^*

†Equal contribution to this work.

*Correspondence author: Shi Chen, Lianrong Wang

Full address: 115 Donghu Road, Wuchang District, Wuhan, China

E-mail: shichen@whu.edu.cn; lianrong@whu.edu.cn

This file includes:

Figures S1 to S3

**Figure S1. (A-B)** Protein level of the full-length APP in the endosomal fraction isolated from rat hippocampus. Equal amount protein of endosomal fraction was loaded in each lane. Three-month-old male rats of *WT* and *SorL1 KO* rats were used for subcellular fractionation through density gradient centrifugation. Three independent experiments were performed with *WT* (n=5) and *SorL1 KO* (n=5) rats. Data from individual rats are shown by dots, and the mean ± SEM values are presented in a bar chart in B). **(C-D)** Purity of the subcellular fractions isolated from the rat hippocampus. The lysate of the subcellular fractions was examined by detecting EEA1 for endosomes, Lamp1 for lysosomes, Histone 3 (H3) for the nucleus and GAPDH for the cytoplasm. **(F-G)** Abundance of the autophagy regulator P62 in the rat hippocampus. Data from individual rats are shown by dots, and the mean ± SEM values are presented in a bar chart in G). Three independent experiments were performed with three rats per genotype. *WT* (n=5) and *SorL1* *KO* (n=6) rats were detected. **(H)** Lactic acid level in cardiac puncture blood of rats. *WT* (n=7) and *SorL1* *KO* (n=6) rats were detected. **(I-J)** Expression of MCT1 in hippocampal lysates from *WT* (n=7) and *SorL1 KO* (n=6) rats. Data from individual rats are shown by dots, and the mean ± SEM values are presented in a bar chart in J). Three independent experiments were performed. Two-tailed t tests were performed, with ***p<0.001 indicating significant differences and *n.s.* indicating no significance.





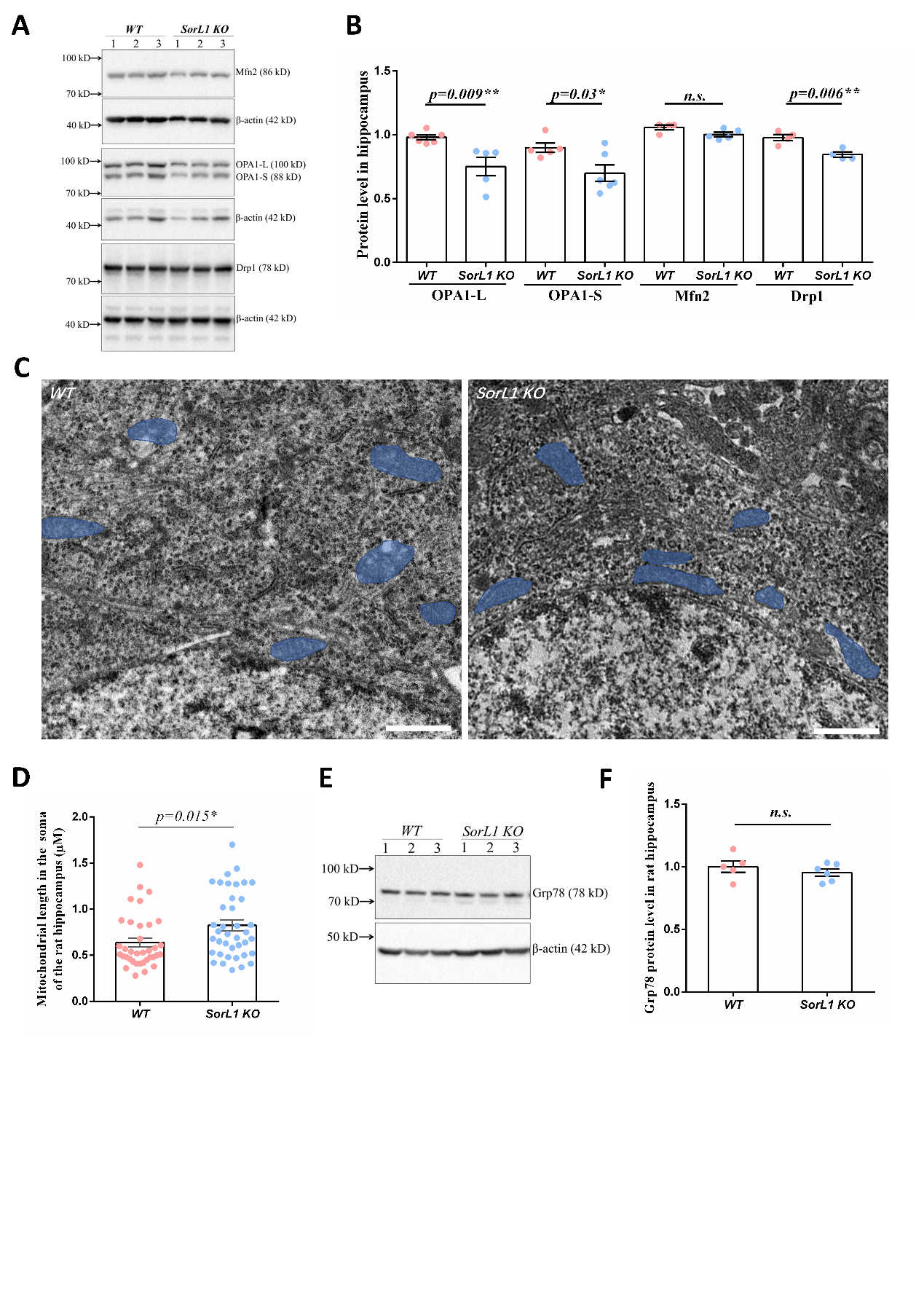


**Figure S2. (A-B)** Expression of mitochondrial fission/fusion regulators in hippocampal lysates from *WT* and *SorL1 KO* rats. Data from individual rats are shown by dots, and the mean ± SEM values are presented by in a bar chart in B). Three independent experiments were performed. For the OPA1 assay, *WT* (n=6) and *SorL1 KO* (n=6) rats were used. For the Mfn2 assay, *WT* (n=4) and *SorL1 KO* (n=5) rats were used. For the Drp1 assay, *WT* (n=4) and *SorL1 KO* (n=4) rats were used. **(C)** Mitochondrial morphology in the soma of the hippocampal neuron (shaded blue). Scale bar = 1 μm. **(D)** Mitochondrial morphology assay. The length between the longest two ends of each mitochondrial particle in TEM photos was measured and indicated with a dot. The bar chart is presented as the mean ± SEM with ten photos of three three-month-old male rats of each genotype. Two-tailed t tests were used. **p* < 0.05 indicates a significant difference. **(E-F)** The expression of Grp78 in hippocampal lysates from *WT* (n=5) and *SorL1 KO* (n=6) rats. Data from individual rats are shown by dots, and the mean ± SEM values are presented in a bar chart in F). Two-tailed t tests were performed, with *p<0.05, **p<0.01 indicating significant differences and *n.s.* indicating no significance.





**Figure S3. (A-B)** Cumulative distance and mean velocity of the rats in the MWM probe test. The data of individual rats are indicated by dots, and the mean ± SEM for each genotype is presented. *WT* (n=8), *SorL1 KO* (n=8) and *DKO* (n=6) rats were used. One-way AVONA were used, and *n.s.* indicates no significant differences. **(C-D)** Expression of TMEM175 in hippocampal lysates from *WT* and *SorL1 KO* rats. Data from individual rats are shown by dots, and the mean ± SEM values are presented in a bar chart in D). Three independent experiments were performed. The *WT* (n=5) and *SorL1 KO* (n=7) male rats of three-month-old were assayed. Two-tailed t tests were performed, with **p<0.01 indicating significant differences. **(E-F)** Expression of mitochondrial biogenesis regulators in hippocampal lysates from *WT* and *SorL1 KO* rats. The stray band is indicated by the asterisk. Data from individual three-month-old male rats are shown by dots, and the mean ± SEM values are presented in a bar chart in F). Three independent experiments were performed. For the PGC1α assay, *WT* (n=8) and *SorL1 KO* (n=8) rats were used. For NRF2 and TFAM assays, *WT* (n=5) and *SorL1 KO* (n=5) rats were analyzed. Two-tailed t tests were performed, with **p<0.01 indicating significant differences.
