# Supplementary material for: Endo‐Lysosomal Network Disorder Reprograms Energy Metabolism in SorL1‐Null Rat Hippocampus
Source: Adv Sci (Weinh). 2024 Sep 3;11(41):2407709. doi: 10.1002/advs.202407709 (PMC11538633; doi:10.1002/advs.202407709)

ADVANCED SCIENCE
Open Access

Supporting Information

for *Adv. Sci.*, DOI 10.1002/advs.202407709

Endo-Lysosomal Network Disorder Reprograms Energy Metabolism in *SorL1*-Null Rat Hippocampus

Yajie Wang, Yuting Yang, Ying Cai, Ayikaimaier Aobulikasimu, Yuexin Wang, Chuanwei Hu, Zhikang Miao, Yue Shao, Mengna Zhao, Yue Hu, Chang Xu, Xinjun Chen, Zhiqiang Li, Jincao Chen, Lianrong Wang and Shi Chen**

S1A

Lane1-3: Endosomal fraction of WT hippocampus
Lane4-6: Endosomal fraction of SorL1 KO hippocampus

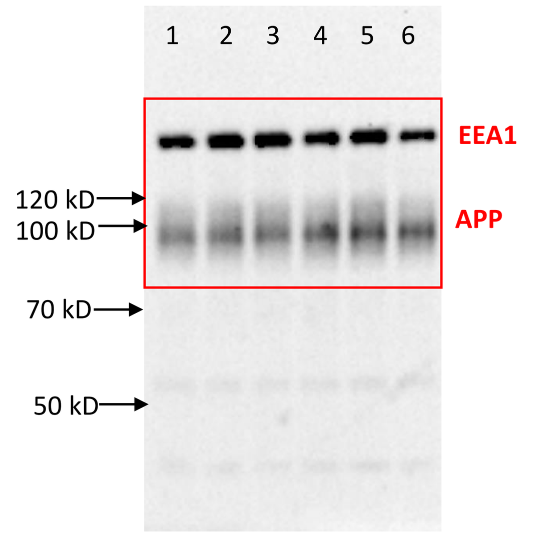

S1C

Lane1: Total
Lane2: Endosomal fraction

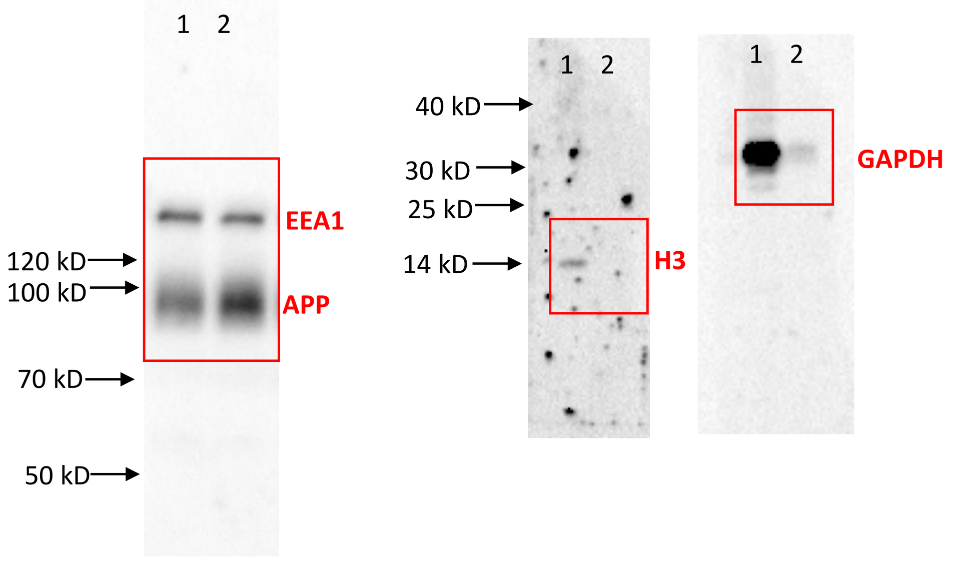

S1D

Lane1: Total
Lane2: Lysosomal fraction

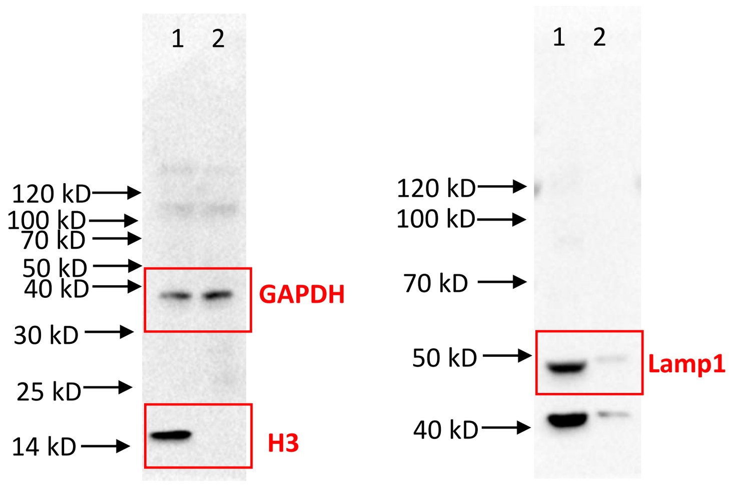

S1E

Lane1: Total
Lane2: Cytoplasmic fraction
Lane3: Nuclear fraction

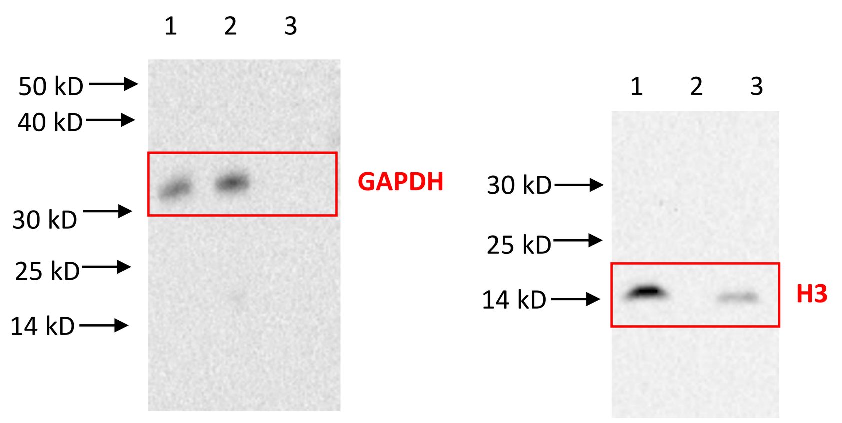

S1F

Lane1-3: WT
Lane4-6: SorL1 KO

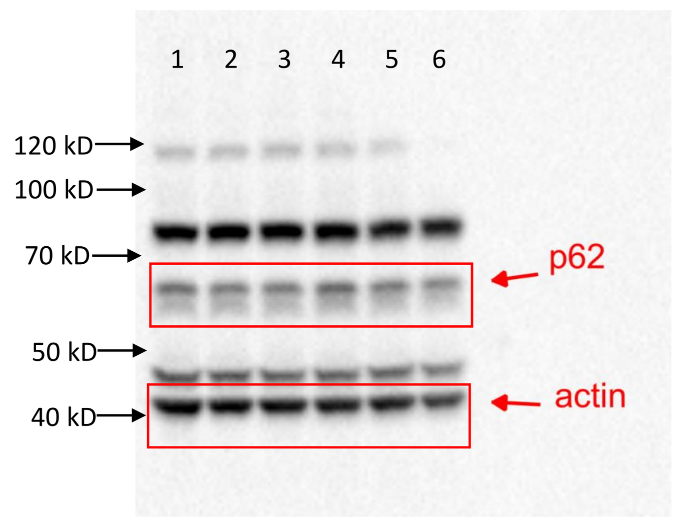

S1I

Lane1-3: WT
Lane4-6: SorL1 KO

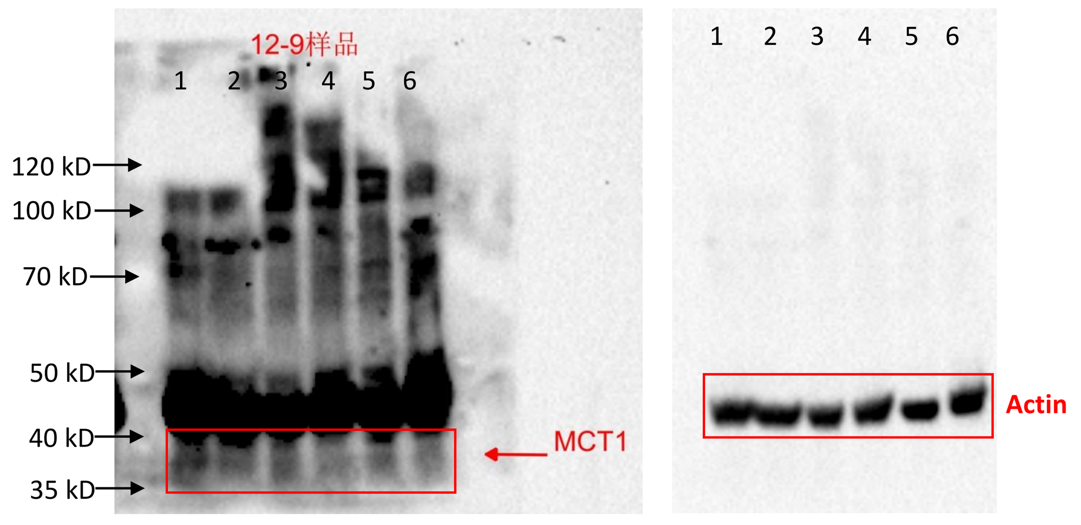

S2A

Lane1-3: WT
Lane4-6: SorL1 KO

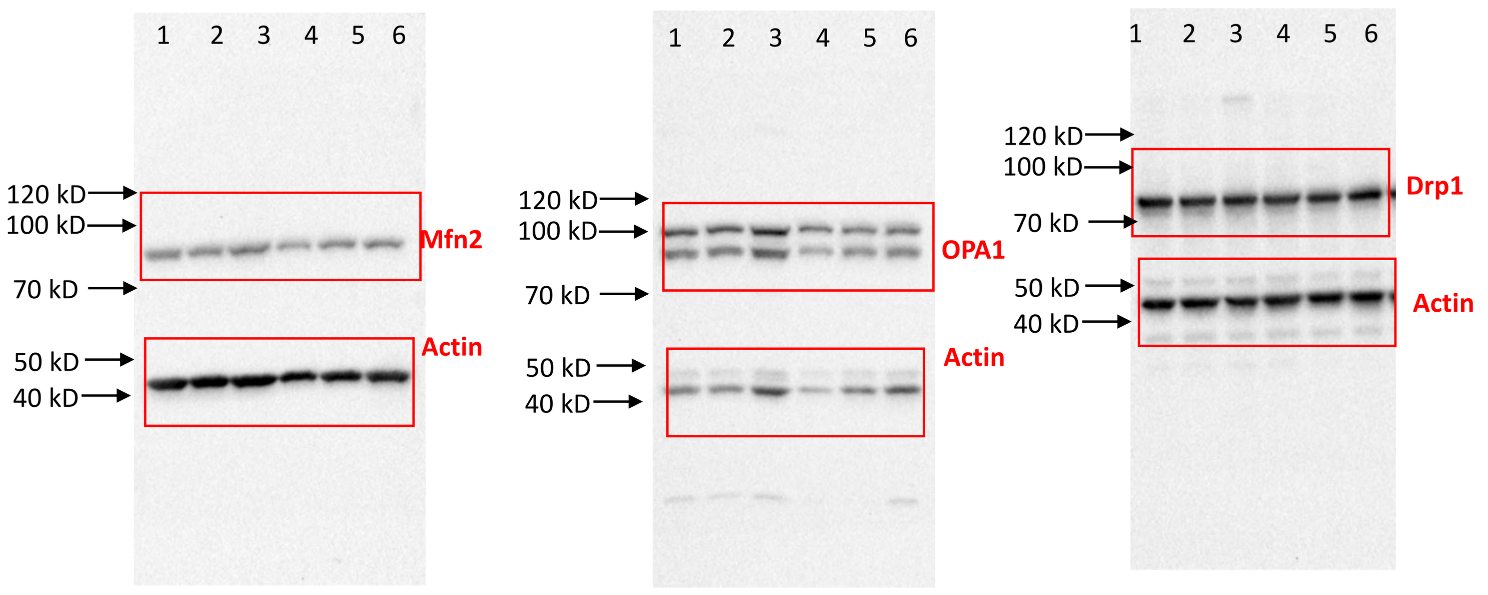

S2E

Lane1-3: WT
Lane4-6: SorL1 KO

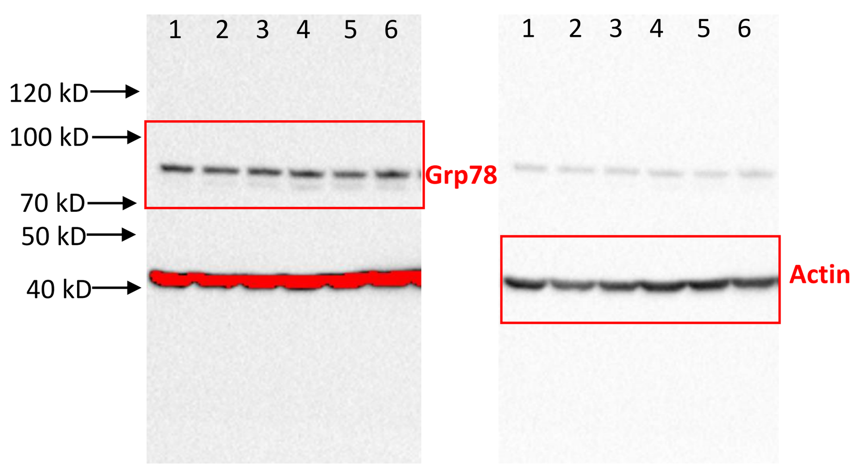

S3C

Lane1-3: WT
Lane4-6: SorL1 KO

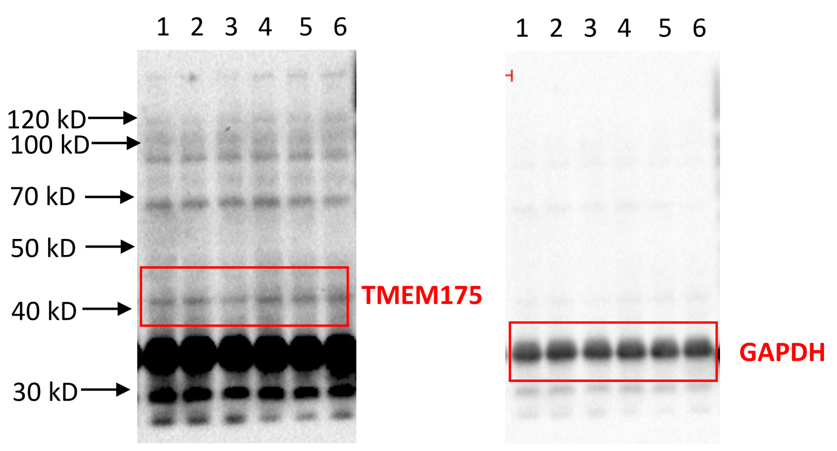

S3E

Lane1-3: WT
Lane4-6: SorL1 KO

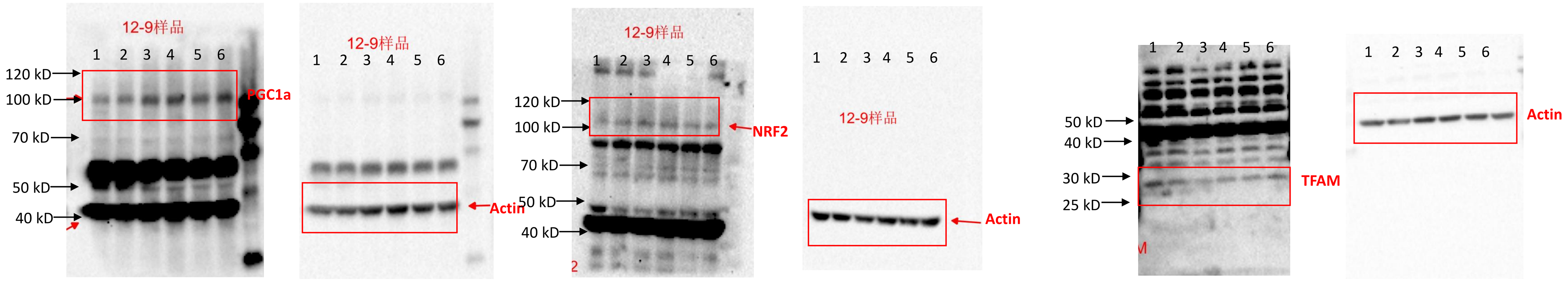

Supplement: Supplementary file 2 — Supporting Information [file ADVS-11-2407709-s001.pdf]
